# Genome-wide Association Analysis Tracks Bacterial Leaf Blight Resistance Loci In Rice Diverse Germplasm

**DOI:** 10.1186/s12284-017-0147-4

**Published:** 2017-03-21

**Authors:** Christine Jade Dilla-Ermita, Erwin Tandayu, Venice Margarette Juanillas, Jeffrey Detras, Dennis Nicuh Lozada, Maria Stefanie Dwiyanti, Casiana Vera Cruz, Edwige Gaby Nkouaya Mbanjo, Edna Ardales, Maria Genaleen Diaz, Merlyn Mendioro, Michael J. Thomson, Tobias Kretzschmar

**Affiliations:** 1 Plant Breeding, Genetics and Biotechnology Division, International Rice Research Institute, Los Baños, Laguna Philippines; 2 Crop, Soil, and Environmental Science, University of Arkansas, Fayettevile, AR USA; 3 Crop Protection Cluster, University of the Philippines Los Baños, College, Laguna, Philippines; 4 Institute of Biological Sciences, University of the Philippines Los Baños, College, Laguna, Philippines; 5 Department of Soil and Crop Sciences, Texas A&M University, College Station, TX USA

**Keywords:** Genome-wide association, *Oryza sativa*, *Xanthomonas oryzae*, bacterial blight (BB), disease resistance, genotyping-by-sequencing (GBS), SNPs

## Abstract

**Background:**

A range of resistance loci against different races of *Xanthomonas oryzae pv. oryzae* (*Xoo*), the pathogen causing bacterial blight (BB) disease of rice, have been discovered and characterized. Several have been deployed in modern varieties, however, due to rapid evolution of *Xoo*, a number have already become ineffective. The continuous “arms race” between *Xoo* and rice makes it imperative to discover new resistance loci to enable durable deployment of multiple resistance genes in modern breeding lines. Rice diversity panels can be exploited as reservoirs of useful genetic variation for bacterial blight (BB) resistance. This study was conducted to identify loci associated to BB resistance, new genetic donors and useful molecular markers for marker-assisted breeding.

**Results:**

A genome-wide association study (GWAS) of BB resistance using a diverse panel of 285 rice accessions was performed to identify loci that are associated with resistance to nine *Xoo* strains from the Philippines, representative of eight global races. Single nucleotide polymorphisms (SNPs) associated with differential resistance were identified in the diverse panel and a subset of 198 *indica* accessions. Strong associations were found for novel SNPs linked with known bacterial blight resistance *Xa* genes, from which high utility markers for tracking and selection of resistance genes in breeding programs were designed. Furthermore, significant associations of SNPs in chromosomes 6, 9, 11, and 12 did not overlap with known resistance loci and hence might prove to be novel sources of resistance. Detailed analysis revealed haplotypes that correlated with resistance and analysis of putative resistance alleles identified resistant genotypes as potential donors of new resistance genes.

**Conclusions:**

The results of the GWAS validated known genes underlying resistance and identified novel loci that provide useful targets for further investigation. SNP markers and genetic donors identified in this study will help plant breeders in improving and diversifying resistance to BB.

**Electronic supplementary material:**

The online version of this article (doi:10.1186/s12284-017-0147-4) contains supplementary material, which is available to authorized users.

## Background

Bacterial blight (BB) disease, caused by *Xanthomonas oryzae pv. oryzae* (*Xoo*), is one of the most widespread and economically important diseases of rice. In Asia, continuous rice cultivation has resulted in highly virulent *Xoo* races that cause yield reductions of up to 50% in certain environments (Mew et al. 1993). Diversity of *Xoo* was characterized across Asia and revealed five distinct clusters composed of seven pathotypes (Adhikari et al. 1995). Pathotype 1 was widespread in Malaysia, Philippines and Korea. *X* *oo* populations in Indonesia mainly belonged to pathotype 3, while pathotypes 4 and 6 were common in Nepal, India, and the Philippines (Adhikari et al. 1995). A more recent comparative genomic study on the 10 major *Xoo* races from the Phillipines clustered them in three major lineages, PX-A (Races 1, 3c, 4, 9b and10), PX-B (Races 2, 5, 7 and 8) and PX-C (Race 6, strain PXO99) (Quibod et al. 2016). Though these were distinct from African lineages, they were suggested to be conserved across Asia. Historical data furthermore revealed two major shifts in the distributions of *Xoo* races in the Philippines. While race 1 was predominant in early 1970s, it was replaced by race 2 in the 1980s-1990s. Since 1992 race 9b has expanded and become the predominant race in the 2010s (Quibod et al. 2016).

As distributions are changing and new virulent *Xoo* races are emerging, host plant resistance remains a key component of BB disease management (Adhikari et al. 1998). Considerable efforts have been mounted towards breeding for resistance against *Xoo.* Genetic mapping studies for BB resistance have led to the identification of 40 *Xa* resistance loci (Nino-Liu et al. 2006; Verdier et al. 2011; Bhasin et al. 2012; Zhang et al. 2015; Kim et al. 2015), most of which were directly derived from *Oryza sativa* accessions. Nine genes have been cloned, namely *Xa1*, *Xa3*/*Xa26*, *xa5*, *Xa10*, *xa13*, *Xa21*, *Xa23*, *xa25*, and *Xa27* (Nino-Liu et al. 2006; Liu et al. 2011; Tian et al. 2014; Wang et al. 2014b).

Five BB resistance loci (*xa5, Xa7, xa13, xa24 and xa34*) originated from *aus* genotypes (Sidhu et al. 1978; Ogawa et al. 1987; Khush and Angeles 1999; Chen et al. 2011). Among these loci, the recessive *xa5*, encoding the gamma subunit of transcription factor IIA, is the only gene to be linked with resistance to Race 9 and furthermore confers broad-spectrum resistance to Philippine races 1, 2, 3, 5, 7, 8, and 10 (Iyer and McCouch 2004; Nino-Liu et al. 2006). *Xa7* is a dominantly inherited TAL effector R gene that recognizes TAL effector proteins (Römer et al. 2009) and confers to race 1 (PXO61), race 2 (PXO86), and race 3 (PXO79) (Sidhu et al. 1978). Resistance to race 6 is recessively conferred by *xa13, a* nodulin MtN3 gene (Ogawa et al. 1987; Chu et al. 2006a, b) and *xa24* (Khush and Angeles 1999).

Ten BB resistance loci (*Xa2*, *Xa4*, *Xa11*, *Xa14*, *Xa16*, *Xa18*, *xa25*, *Xa26*, *xa28* and *Xa39*) originated from *indica* donors (Verdier et al. 2011; Zhang et al. 2015). *Xa4* is a dominant resistance locus originally identified from TKM6 (Ogawa et al. 1986). It has been widely used in rice breeding programs (Mew 1987) that led to the emergence of new virulent race 2 and race 3 strains (Mew et al. 1992). Despite its breakdown, *Xa4* acts as a recessive QTL, exhibiting epistatic or additive effects when combined with other resistance genes (Li et al. 2001). *Xa14* is a dominant resistance locus against race 5 which was found in Taichung Native 1 (TN1) by Taura et al. (1987). The recessive *xa25* from Minghui 63 (Chen et al. 2002), encodes a nodulin MtN3 family protein essential for reproductive development and rice-*Xoo* interaction (Yuan and Wang 2013), and confers a high level of resistance to race 9a (PXO339) (Liu et al. 2011)*.*

Nine dominant *Xa* loci were derived from wild relatives of rice (*Xa21*, *Xa23*, *Xa27*, *Xa29*, *Xa30*, *Xa32*, *Xa35*, *Xa38* and *Xa40*) (Song et al. 1995; Zhang et al. 2001; Gu et al. 2004; Tan et al. 2004; Cheema et al. 2008; Zheng et al. 2009; Guo et al. 2010; Bhasin et al. 2012; Kim et al. 2015). Notably *Xa21* encoding a receptor kinase-like protein involved in the recognition of pathogen effectors and the activation of defense response (Song et al. 1995) was the first cloned R gene for BB resistance. Originating from *Oryza longistaminata*, it has been widely introduced to popular varieties (Verdier et al. 2011), conferring broad-spectrum resistance to Philippine *Xoo* races (Ikeda et al. 1990; Khush, et al. 1990).

The continuous mono-cropping and deployment of rice cultivars with a narrow genetic base has provided high selection pressure for emerging virulent strains. Cultivars with single major genes for resistance are prone to resistance breakdown due to pathogen variation and evolution. Thus, pyramiding of resistance genes into individual breeding lines is a strategy to provide more durable resistance (Gnanamanickam et al. 1999; McDowell and Woffenden 2003). Though pyramiding of known loci is a promising approach for disease management, novel sources of resistance are required to keep the upper hand in the continuous plant-pathogen “arms race”. Thus, the search for new genes is crucial to reinforce host resistance breeding. Rice has a wide range of genetic diversity and global germplasm collections, serving a rich source of novel resistance loci. Useful genes for biotic stress resistance were classically identified through biparental mapping populations using landraces and wild ancestors as donors (Ikeda et al. 1990).

More recently, the advent of high-density molecular marker platforms in conjunction with carefully assorted diversity panels has enabled the exploration of agronomic traits through GWAS and allele mining. GWAS facilitates the discovery of novel alleles and allele combinations that are useful in crop improvement (Zhu et al. 2008; Tung et al. 2010). Association studies on sheath blight (Jia et al. 2012) and blast (Wang et al. 2014a, b) disease resistance in rice to identify resistance loci and novel functional candidate genes exemplifies the power of GWAS. With advances in genomics tools, this study aims to identify resistance loci associated with BB resistance and useful SNP markers to track these loci, and determine new genetics donors through GWAS.

## Results

### Genetic Diversity And Population Structure

Based on genetic diversity, 285 accessions were selected as initial core panel from an original collection of 380 genotypes (Additional file 1: Table S1). The panel comprised 208 breeding lines and varieties, and 77 landraces. Phylogenetic analysis featured five distinct clusters, namely three clusters of *indica*, one cluster for *aus*, and a cluster containing *japonica* and *aromatic* rice varieties (Additional file 2: Figure S1a). Mean genetic distance within the three *indica* clusters was 0.16 while the *aus* and *japonica*/*aromatic* clusters displayed 0.24 and 0.09 mean genetic distance, respectively. Population differentiation (*Fst*) of *indica* genotypes from *aus* genotypes is 0.13 while 0.20 from *japonica*/*aromatic.* Overall, differentiation based on global unbiased *Fst* of 0.10 across the five clusters was determined. Principal component analysis separated the 285 diverse panel into five subgroups. The *indica* subset (198 genotypes) was differentiated by PC = 3 (Additional file 2: Figure S1d).

### BB Resistance Screening

**Fig. 1:**
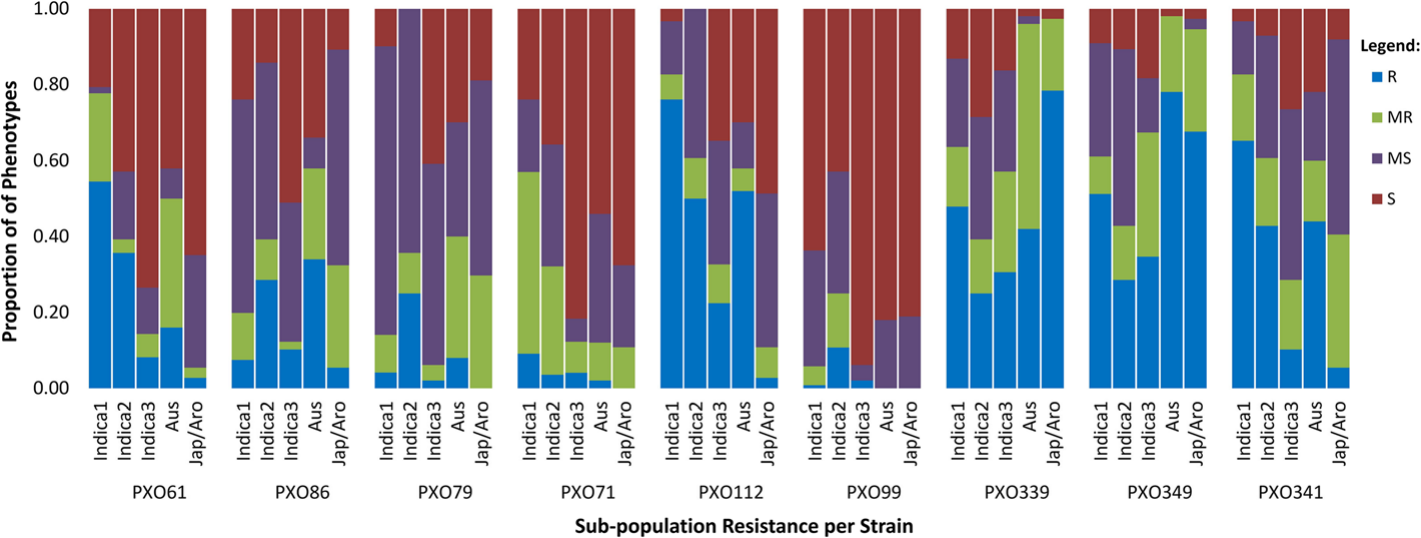
Sub-population resistance of the 285 diverse germplasm to nine representative strains of Xanthomonas oryzae pv. oryzae

Nine Philippine strains (PXO) representing eight different races of *Xanthomonas oryzae pv. oryzae* (Table 1) were used to screen the 285 genotypes and a wide range of disease resistance was observed (Fig. 1; Additional file 1: Table S1). A set of 17 near isogenic lines (NILs) in IR24 background, having single or two to four pyramided *Xa* genes (Huang et al. 1997) were included in the panel to serve as controls of known disease reactions. Results of the BB resistance screening mostly conformed to the expected reactions of the NILs. Out of the 285 genotypes, IRBB59, IRBB60, IRBB66, CBB23, IR09F154, IR08N134, and IR72890-81-3-2-2 exhibited resistance to all nine *Xoo* races. Several genotypes exhibited resistance against the most virulent *Xoo* race, Race 6 (PXO99), namely IRBB59, IR09F154, IRBB60, IRBB23, CBB23, IRBB66, IR08N134, IR 72890-81-3-2-2, IR08N104, IR09N495, IR06A147, and PSB Rc88 (Additional file 1: Table S1). Some of these resistant genotypes are known to possess *xa13* and *Xa21* or combination of three to four *Xa* genes, conferring resistance to PXO99 (Huang et al. 1997). CBB23 and IRBB23 possess the *Xa23* gene that confers broad resistance at all developmental stages of rice and high resistance to PXO99 (Table 1) (Zhang et al. 2001).

**Table 1:** Nine strains of *Xanthomonas oryzae* pv. *oryzae* used in this study

Race	Strain^a^	Genes Conferring Resistance^b^
1	PXO61	*Xa4,xa5,Xa7,Xa21,Xa22,Xa23,Xa26,Xa32*
2	PXO86	*xa5,Xa7,Xa10,Xa21,Xa23,Xa27,xa28*
3b	PXO79	*xa5,Xa7,Xa21,Xa23*
4	PXO71	*Xa4,Xa21,Xa23,xa24,Xa32*
5	PXO112	*Xa4,xa5,Xa7,xa8,Xa10,Xa14,Xa21,Xa23,Xa27,xa28,Xa31,Xa32,Xa35*
6	PXO99	*xa13,Xa21,Xa23,xa24,Xa30,Xa32*
9a	PXO339	*xa5,Xa21,Xa23,xa25,Xa32,Xa35*
9b	PXO349	*xa5,Xa21,Xa23,Xa25*
10	PXO341	*Xa4,xa5,Xa7,Xa21,Xa23*

^a^Philippine representative strains to specific *Xoo* races

^b^Naturally occuring *Xa* genes known to confer resistance against specific *Xoo* races based on review given by Nino-Liu et al. (2006) and Verdier et al. (2011)

Rice varieties belonging to the *aus* group also displayed high levels of resistance against *Xoo*. Aus 299, Dular, Aus Bak Tulsi, Ase Pulu Jawa, Aus 298, Makalioka 34, Kalimekri 77-5, DV 86, Chengri, Aus 307, Khaiyan, and DV85 are *aus* genotypes that were found to consistently exhibit moderate to high resistance across eight *Xoo* races. Notably most of the *aus* genotypes (96%) in this study exhibited resistance to both strains of race 9 (PXO339 and PXO349) (Fig. 1; Additional file 1: Table S1).

### Genome-wide Association Analysis

The 285 genotypes in the diverse panel exhibited a distinct population structure (Additional file 2: Figure S1) which can cause false positives if not corrected (Zhu et al. 2008). Initial results using unified Mixed Model that incorporate principal component Analysis (PCA) showed ambiguous peaks in the Manhattan plots that could have been an effect of residual population structure not fully corrected by Q + K (data not shown). Thus, 37 *japonica*/*aromatic* genotypes were excluded. A total of 248 samples composed of *indica* and *aus* genotypes (henceforth “*indica/aus* panel”) were retained for subsequent genome wide analysis that was performed using 40,840 SNPs and phenotyping information for nine *Xoo* strains. Furthermore, separate analyses for 198 *indica* genotypes (henceforth “*indica* subset”) based on 40,396 SNPs were conducted. Manhattan plots for the two separate analyses showed similar trends between the *indica/aus* panel and the *indica* subset except for PXO86, PXO99 and PXO341 (Additional file 3: Figure S2 and Additional file 4: Figure S3). Peaks exclusively detected in the *indica/aus* panel were noticeable in the centromere region of chromosome 10 for PXO86 (Additional file 3: Figure S2b and Additional file 4: Figure S3b), and short arm of chromosome 5 and long arm of chromosome 12 for PXO341 (Additional file 3: Figure S2i and Additional file 4: Figure S3i). However, more distinct peaks for PXO99 were observed in the analysis of the *indica* subset and the detection of the *xa13* region in chromosome 8 (Additional file 3: Figure S2f and Additional file 4: Figure S3f).

#### Significant Hits Overlapping Known Resistance Genes

GWAS identified SNPs that were consistently associated with resistance against several strains. Known genes and loci (*Xa*) conferring resistance to specific *Xoo* races (Table 1) were overlaid with the Manhattan plots (Additional file 3: Figure S2 and Additional file 4: Figure S3). Among the known *Xa* loci, the regions of *Xa4, xa5, Xa7, xa13, Xa14*, *Xa21, and xa25* (Wang et al. 2001; Blair et al. 2003; Chen et al. 2008; Chu et al. 2006a, b; Bao et al. 2010; Song et al. 1995; Chen et al. 2002) were identified as overlapping with significant SNPs in this study (Additional file 3: Figure S2, Additional file 4: Figure S3 and Additional file 5: Table S2).

**Fig. 2:**
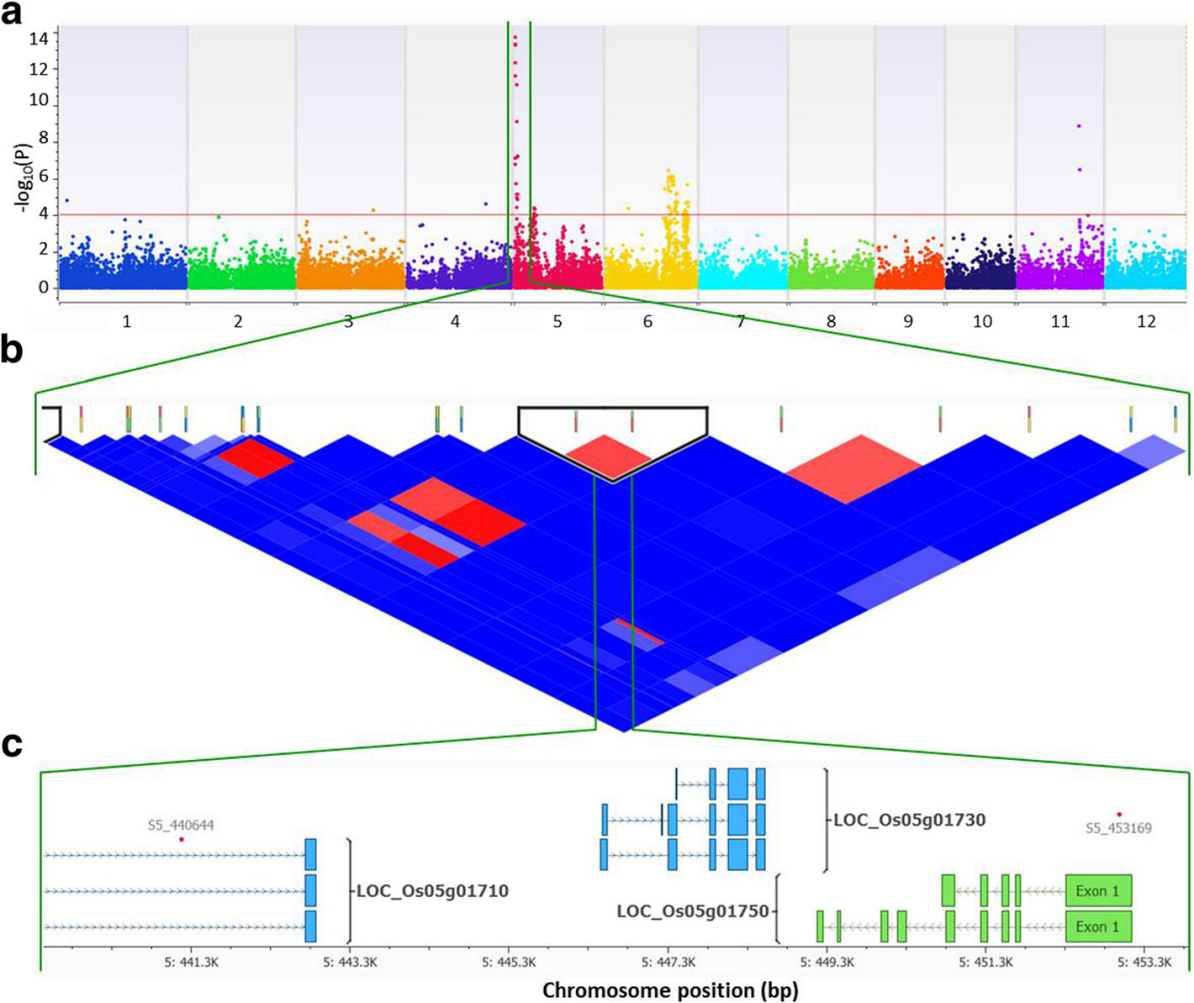
Highly associated SNPs with resistance to Xoo Race 3b (PXO79) and overlapping the *xa5* gene. **a** Manhattan plot based on Efficient Mixed-Model Association eXpedited Model (EMMAX) where x-axis shows the SNPs along each chromosome and y-axis is the − log10 (*P*-value) for the association. Significant SNPs are those beyond the red line having *P*-value < 1 × 10^−5^. **b** LD (r^2^) and haplotype blocks based Expectation Maximization (EM) algorithm. **c** Significant SNP S5_440644 overlapping the region of LOC_Os05g01710, a transcription initiation factor IIA gamma chain

Significant SNP loci exclusively associated with resistance to PXO112 in the *indica* panel (Additional file 5: Table S2) clustered in the flanking region of *Xa14* (Bao et al. 2010) that ranges from 30,063,575 bp-32,807,203 bp in the long arm of chromosome 4 (Additional file 4: Figure S3e). On the other hand, SNP loci located in the short arm of chromosome 5 namely, S5_440644 and S5_453169 were highly associated with resistance to all seven strains of *Xoo* (Additional file 3: Figure S2, Additional file 4: Figure S3 and Additional file 5: Table S2). S5_453169 was identified by MLMM (*p*-value of 5.95^−17^) to be a cofactor for resistance against PXO61 and PXO349. S5_440644 overlaps with a transcription initiation factor IIA gamma chain (LOC_Os05g01710), a similar gene that encodes the recessive *xa5* gene (Fig. 2).

On the long arm of chromosome 6 a cluster of SNPs was associated with resistance against PXO79 (Additional file 5: Table S2). Among them S6_28100960 accounted for 11% of the phenotypic variation and was approximately 68 kb away from the ID7 marker for *Xa7* (Zhang et al. 2009a). Furthermore, S6_28100960 was a cofactor for resistance to PXO79 (race 3b) in MLMM, confirming previous reports that *Xa7* confers differential resistance against race 1, 2, 3, and 5 during flowering and later growth stages (Lee et al. 2003). A congruent peak for PXO61 and PXO86 was just below the significance threshold (Additional file 3: Figure S2 and Additional file 4: Figure S3).

For PXO99 (race 6), pronounced differences in the Manhattan plots were found between association analyses for the *indica/aus* panel and the *indica* subset. Significant SNPs clustering the region of the recessive resistance gene *xa13* were detected exclusively in the *indica* subset (Additional file 4: Figure S3f and Additional file 5: Table S2). Two distinct peaks were observed flanking the actual physical position of *xa13* (26,725,898-26,728,795 bp) in the long arm of chromosome 8 (Chu et al. 2006a, b). Both peaks (25,954,303 -26,044,528 bp and 27,223,555-27,663,433 bp) were both approximately 500 kb away from *xa13* (DQ421394). Despite *xa13* having originated from an *aus* variety BJ1 (Ogawa et al. 1987), association analysis of PXO99 resistance in the *indica/aus* panel (50 of which are *aus*) revealed no significant associations at the long arm of chromosome 8 (Additional file 3: Figure S2f). Instead, distinct peaks in the short arm of chromosome 5 (*xa5*) and in the long arm of chromosome 11 (*Xa21*) (Additional file 3: Figure S2f) were associated with resistance to PXO99. It was pointed out by Li et al. (2001) that *xa13* strongly interacts with other *Xa* genes such as *xa5*, *Xa4* and *Xa21*. Besides, other genes masking *xa13*, allele frequency and effects of population structure not accounted for by the Q and K matrices may have affected associations in the *indica*/*aus* panel.

**Fig. 3:**
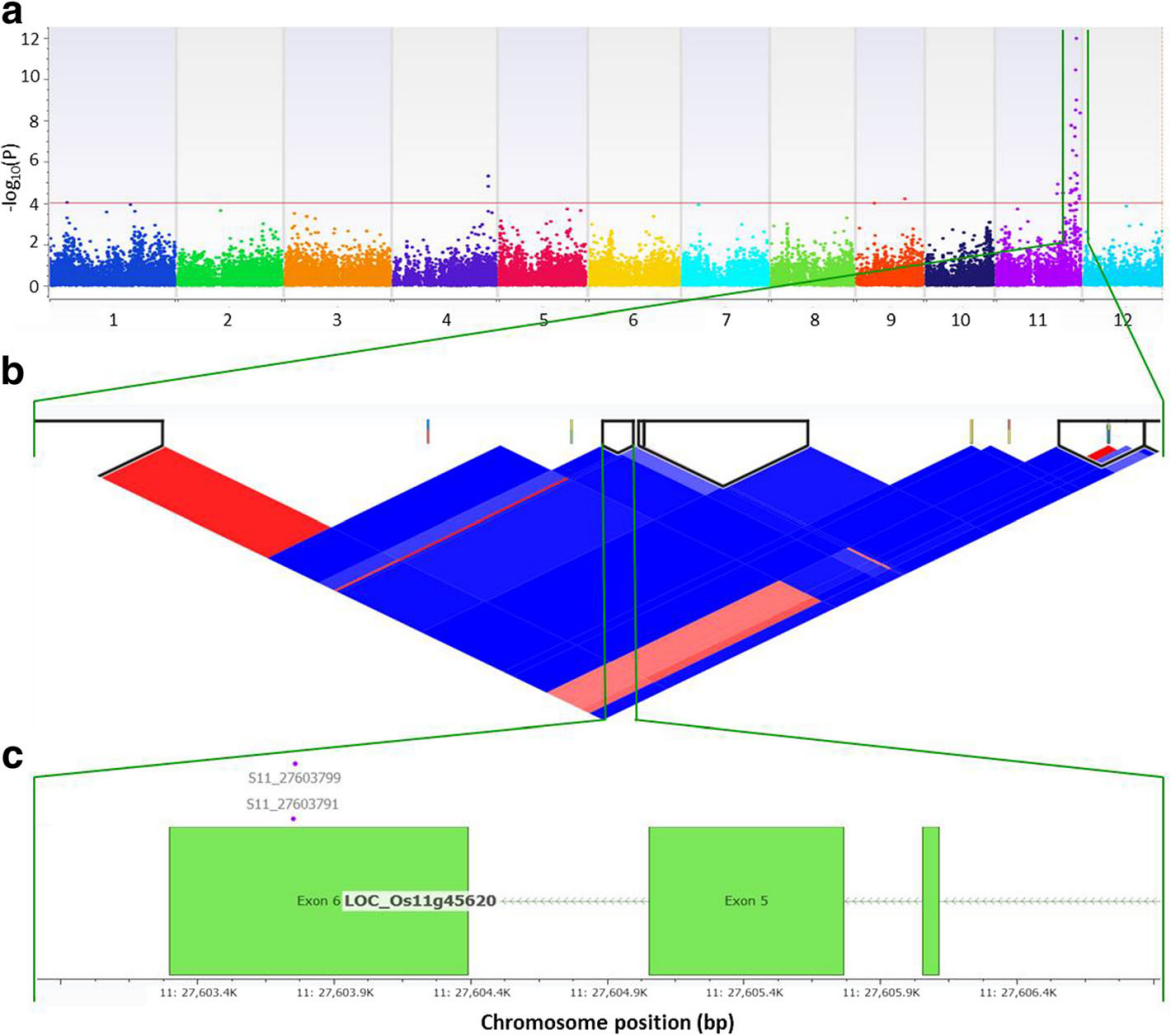
Highly associated SNPs with resistance to *Xoo* Race 4 (PXO71) and flanking *Xa4* gene. **a** Manhattan plot based on Efficient Mixed-Model Association eXpedited Model (EMMAX) where x-axis shows the SNPs along each chromosome and y-axis is the − log_10_ (*P*-value) for the association. Significant SNPs are those beyond the red line having *P*-value < 1 × 10^−5^. **b** LD matrix (r^2^) and haplotype blocks based on Expectation Maximization (EM) algorithm. **c** Significant SNPs (S11_27603791 and S11_27603799) overlapping the exon region of LOC_Os11g45620 (rust-resistance protein *Lr*21), flanking the *Xa4* locus

Among the twelve chromosomes of rice, chromosome 11 is well known as a hotspot of BB resistance genes (Khush and Brar 2001; Khush and Virk 2005; Ghazi et al. 2009). In the *indica/aus* panel S11_21190115 and S11_21246561, proximal to *Xa21* (21,273,533 -21,277,443 bp) (Ronald et al. 1992; Song et al. 1995), were highly associated with resistance to PXO61, PXO86, PXO79, PXO112, PXO99, and PXO349 (Additional file 5: Table S2). Furthermore, S11_21190115 SNP was identified as a cofactor for PXO99 resistance in the *indica* subset. Six significant SNPs (S11_27007623 to S11_27831021) were flanking the region of the RM224 marker (27,673,250-27,673,372 bp) linked to the *Xa4* region (Wang et al. 2001; Sun et al. 2003) (Fig. 3 and Additional file 5: Table S2). Among them, S11_27603799 accounted for 29% of phenotypic variance and was a cofactor for resistance to PXO61, PXO71, PXO112 and PXO341 based on MLMM (*p*-value of 4.66^−25^). The three most downstream SNPs (S11_27672709, S11_27677853 and S11_27831021) overlapped with the recently fine-mapped *Xa4* region containing NBS-LRR and WRKY gene family members (Hur et al. 2016). The resistance reactions associated with these SNPs including 2 additional upstream SNPs (S11_27007623 and S11_27007628) agreed with earlier reports for *Xa4* (Nino-Liu et al. 2006). Association analyses of resistance to PXO339 (race 9a) and PXO349 (race 9b) showed similar patterns in the Manhattan plots (Additional file 3: Figure S2 and Additional file 4: Figure S3). A wide peak covering 13 Mb to 17 Mb in the centromeric region of chromosome 12 was observed in the association with resistance to PXO339 and PXO349 (Additional file 3: Figure S2 and Additional file 4: Figure S3). Two significant SNPs in this region, S12_17305618 and S12_17305619, accounted for 15% of phenotypic variance (Additional file 5: Table S2) and overlapped with *xa25* (LOC_Os12g29220), a gene mapped in the centromeric region of chromosome 12, which confers resistance against PXO339 (Chen et al. 2002).

#### Significant Hits Overlapping Putative Novel Resistance Loci

At a 0.05 Bonferroni-corrected cut-off, and taking local LD into account, 8 peaks, consisting of a total of 33 SNPs on chromosomes 6, 9, 11 and 12 (Additional file 6: Table S3 and Additional file 7: Figure S4), were discovered that did not overlap with previously characterized *Xa* loci (Additional file 3: Figure S2 and Additional file 4: Figure S3). In most cases the minor allele could be associated with resistance (Additional file 6: Table S3), though in some cases the R allele was ambiguous due to high heterozygosity of the SNP.

Notably four out of the 8 peaks, *qPXO79_6-1 to -4*, were specific for resistance against race 3B (PXO79) and clustered between 21.5 Mb and 23.1 Mb on the long arm of chromosome 6. Interestingly *qPXO79_6-1* was specific for the *indica* panel, while *qPXO79_6-2 to -4* were specific for the *indica/aus*. For all 18 SNPs it was the *aus*-enriched allele that was associated with resistance. While the R alleles for *qPXO79_6-4* were minor in the *indica/aus* panel with frequencies of 10–21%, they were highly enriched in the *aus* sub-pool of the *indica/aus* panel with frequencies of 34–82%. Congruently the corresponding R alleles were only found at 5–10% globally in the 3000 Rice Genomes Project (3k genomes), while they were enriched to 37–97% in the *aus* subpool of the 3k genomes.

*qPXO79_6-1* spanned 4 SNPs across 148 kb at 21.4 Mb and included a Putative Serine Carboxypeptidase (LOC_Os06g36570) and an ABC transporter (LOC_Os06g36650) as high priority candidate genes. Serine carboxypeptidases have been implicated in chemical defense against microbes peptide transporter PTR2 (LOC_Os06g38294) as likely candidate gene. PTRs have been shown to be involved in disease resistance (Karim et al. 2007) and chemical defense (Nour-Eldin et al. 2012). Across the 3k genomes, LOC_Os06g38294 featured 11 non-synonymous (NS) SNPs. *qPXO79_6-4* spanning 15 kb at 23 Mb (Mugford et al. 2009) and allele mining across the 3 k genomes for LOC_Os06g36570 revealed 21 non-synonymous NS SNPs. Likewise, ABC transporters (Krattinger et al. 2009) have been shown to contribute to disease resistance and LOC_Os06g36650 contained 21 NS SNPs. The 2 SNPs of *qPXO79_6-2* spanning 69 kb at 21.8 Mb contained a metal cation transporter (LOC_Os06g37010). The 9 SNPs of *qPXO79_6-3* spanning 18 kb at 22.6 Mb contained a peptide transporter PTR2 (LOC_Os06g38294) as likely candidate gene. PTRs have been shown to be involved in disease resistance (Karim et al. 2007) and chemical defense (Nour-Eldin et al. 2012). Across the 3 k genomes LOC_Os06g38294 featured 11 NS SNPs. *qPXO79_6-4* spanning 15 kb at 23.1 Mb consisted of 3 SNPs and featured a receptor-like protein kinase (LOC_Os06g38990) as likely candidate gene.

On the long arm of chromosome 9‚ three SNPs within 40 bp (*qPXO339/349_9-1)* associated with resistance to race 9A and B (PXO339 and PXO349) in both the *indica/aus* and the *indica* panel. They were found at MAFs of 19% and clustered in the first exon of LOC_Os09g38510, which annotated as a purine permease. Purine permeases, implicated in cytokine transporters (Qi and Xiong 2013), have not been described in the context of disease resistance. Linkage of these SNPs with neighboring polymorphisms was weak and high heterozygosity made it difficult to assign the R allele.

Similarly, on the long arm of chromosome 11, *qPXO339/349_11-1,* a very narrow peak of of five SNPs within 60 bp and overlapping with the second exon of a palmitoyltransferase TIP1 (LOC_Os11g34860) associated with race 9A and B (PXO339 and PXO349) resistance in both panels. Palmitoyltransferases have been reported to be involved in pathogen-induced programmed cell death (Saucedo-Garcia et al. 2011). Although linkage with other SNPs in the region was low and heterozygosity high, S11_20426472 was highly significant (*p*-value 3.42^−17^) and determined in MLMM to affect multiple loci underlying resistance.

At 6.4 Mb, a 24 kb peak of four SNPs (*qPXO86_11-1*), was found to be associated with resistance to race 2 (PXO86). *qPXO86_11-1* was limited to the *indica/aus* set, suggesting *aus* lines to be donors. While the R alleles were minor in the diverse set with frequencies of 6–15% they were enriched to 46–71% in the *aus* subpool. In the 3 k genomes, the MAF was only 4–6% globally, but enriched to 21–47% in the *aus*. The region contained one NBS-LRR disease resistance (LOC_Os11g11550) and one NB-ARC domain protein (LOC_Os11g11580). NBS-LRR and NB-ARC-domain proteins are classical resistance proteins involved in pathogen detection and host defense (DeYoung and Innes 2006) in gene-for-gene interactions.

*qPXO79/112/341_12-1* on the long arm of chromosome 12 was associated with resistance to race 3B, 5 and 10. It comprised of 2 tightly linked SNPs separated by 48 kb and was specific for the *indica/aus* set. The rare R alleles were found at only 6% in the *indica/aus* set, but enriched to 25% and 26% in the *aus* subpool. Across the 3 k genomes MAF was 2–3% only but within the *aus* was 26% and 30%. *qPXO79/112/341_12-1* contained three cysteine-rich receptor-like protein kinases, a gene family previously implicated in disease resistance through activation of hypersensitive response upon pathogen recognition (Chen et al. 2004).

### Linkage Disequilibrium, Allele And Haplotype Analysis

Pairwise LD in the regions of *Xa* genes showed a relatively large LD in the *indica* subset (Additional file 8: Figure S5). LD decayed (r^2^ < 0.6) at ~400 kb in the distal end of chromosome 11 (*Xa4* region). A larger LD was observed in the region of *xa13* in chromosome 8 where LD started to decay around 1000 kb. A more extensive LD at ~1500 kb was observed in the putative *xa25* region, found in the centromere of chromosome 12. However, an exception was observed in the *xa5* region which started to decay at around 100 kb. While local LD across *Xa* regions were extensive, average chromosomal LD is weaker and decays within 100–200 kb (Additional file 9: Figure S6).

To identify SNP markers useful for tracking resistance genes, significant SNPs that are flanking or closely linked to a specific *Xa* gene were investigated. For the seven *Xa* genes detected in this study, allele frequency and allele patterns of 24 SNP markers were characterized across the 198 *indica* panel in correlation to disease reaction to the nine *Xoo* strains (Additional file 10: Table S4 and Additional file 11: Table S5).

For the two SNPs flanking *Xa14* (S4_31492731 and S4_31492774), some genotypes carrying the minor alleles displayed resistance to PXO112 and are also known to have other *Xa* genes (Additional file 10: Table S4). Thus, these two SNPs could not be used as marker to track the *Xa14* locus.

The highly significant SNPs for *xa5* (S5_440644 and S5_453169) were found to be in LD (r^2^ = 0.73), forming a haplotype block that was correlated with resistance through haplotype trend regression (Additional file 12: Table S6). All *indica* genotypes having the minor haplotype “AA” exhibited moderate to high resistance across seven *Xoo* strains excluding PXO71 and PXO99. This disease reaction pattern agreed with the expected disease reaction attributed to the *xa5* gene (Nino-Liu et al. 2006). Across 5 R and 10 S lines that are also present in the 3k genomes, the R alleles of S5_440644 (A) and S5_453169 (A) fully segregate with the causal mutation of *xa5* at 437499 (T to A) and the phenotype (Additional file 13: Table S7). Since none of the other NS SNP alleles in the region segregate with phenotype, it suggests that the R alleles for S5_440644 (A) and S5_453169 (A) are indeed indicative of *xa5* and that the SNP at 437499 bp is indeed causal for *xa5*. To validate if *xa5* solely accounted for resistance to PXO339 and PXO349 in *aus*, alleles of the two SNPs (S5_440644 and S5_453169) overlapping *xa5* were further inspected (Additional file 10: Table S4). Among the 50 *aus* genotypes of the *indica/aus* panel, 20 accessions exhibited resistance to the two strains of race 9 but did not have the *xa5* allele (AA allele in DV85 and DV86). Two of these genotypes, Aus299 and Aus307, exhibited resistance to race 9 and three more races in the absence of the putative *xa5* allele. With this, it can be inferred that there might be some other resistance QTL in these *aus* genotypes conferring resistance to race 9 that, due to low allele frequencies, was not detected in the GWAS.

Of the genotypes having the minor alleles of the seven SNPs linked to *Xa7*, 80% exhibited moderate to high resistance against PXO86, PXO79, PXO112, and PXO341 (Additional file 10: Table S4). Indica genotypes having the “A” allele for S6_28100960 always have the minor alleles of the other six SNPs, with average LD (r^2^) of 0.73 among the seven SNPs (Additional file 5: Table S2 and Additional file 8: Figure S5).

Among the ten SNPs flanking the *xa13* region, S8_26013849 was the most significant SNP (*p*-value 1.14^−07^). Genotypes having the homozygous allele “A” for S8_26013849 also have the homozygous minor alleles of the other eight SNPs, exhibiting resistance to PXO99, the most virulent *Xoo* strain in the Philippines (Additional file 10: Table S4). The *xa13* locus encodes a nodulin MtN3 gene, conferring differential resistance which is induced by the recessive allele resulting from mutations in the promoter region (Chu et al. 2006).

The two significant SNPs in chromosome 11 flanking *Xa21* were found useful for tracking *Xa21* gene in the *indica* genotypes. The homozygous minor alleles of S11_21190115 (T) and S11_21246561 (G) corresponded to *indica* genotypes exhibiting *Xa21*-mediated resistance against eight *Xoo* strains (Additional file 10: Table S4). Genotypes having these alleles were BB NILs and breeding lines with pyramided *Xa* genes, explaining the minor allele frequency (MAF) in the *indica* subset and being rare alleles in the 3k genomes (Additional file 11: Table S5).

Contrary to the R alleles of the SNPs flanking or linked to other *Xa* genes, the R alleles (TAT) in the three *Xa4* SNPs (S11_27603791, S11_27603799 and S11_27603835) were major alleles in the *indica* subset (Additional file 10: Table S4 and Additional file 11: Table S5). The *Xa4* gene was broadly deployed in rice breeding programs (Mew 1987), thus explaining the high frequency of the R alleles in the *indica* subset, which was mainly composed of breeding lines and modern varieties. While the R allele frequencies for *Xa4* SNPs were 0.63 in the *indica* subset, their frequencies in *indica* accessions of the 3k genomes were only 0.21 (Additional file 11: Table S5).

For the flanking SNPs of *xa25*, 95% of the genotypes having the “A” allele of S12_17305618 and “T” allele of S12_17305619 displayed resistance to both PXO339 and PXO349 (Additional file 10: Table S4). These results confirmed the earlier reports regarding the differential resistance that *xa25* confer against race 9 of *Xoo* (Chen et al. 2002).

**Fig. 4:**
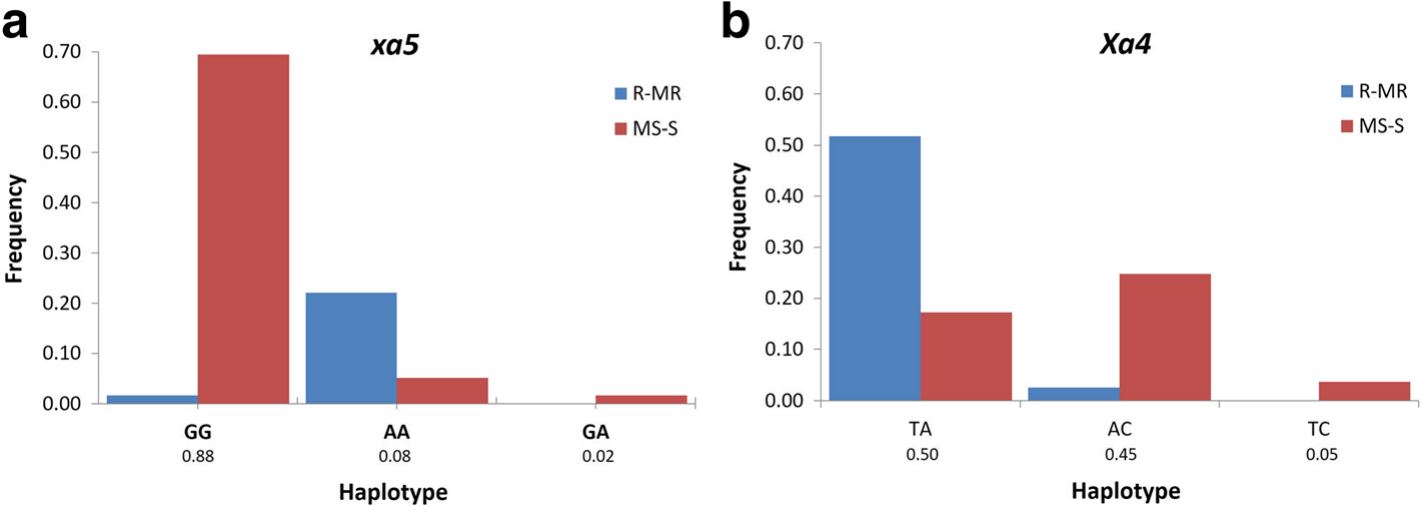
Haplotype blocks overlapping or flanking *Xa* genes. Values below the x-axis indicates the CHM probabilities of the haplotypes (**a**) Haplotype allele frequency of HB1736 overlapping the *xa5* gene in terms of average disease reaction to PXO86, PXO79, and PXO99. **b** Haplotype allele frequency of HB3693 flanking the *Xa4* gene in terms of average disease reaction to PXO61, PXO71, PXO112, PXO99, and PXO341

In many cases a trait of interest cannot be reliably tracked by a single SNP. Haplotype blocks, a pattern of variation across linked SNPs, are often more robust in establishing marker trait associations (Lorenz et al. 2010). Testing associations between phenotypes and haplotypes, instead of single SNPs is thus more appropriate for capturing interactions between SNPs and a QTL (Clark 2004), particularly when multiple alleles are present (Morris and Kaplan 2002). Genome-wide haplotype block detection across the 198 *indica* genotypes resulted in 3,915 haplotype blocks composed of 12,361 SNPs. Based on haplotype trend regression analysis, two significant haplotypes include SNPs that are linked to *Xa4* and *xa5* (Additional file 12: Table S6). As shown in Fig. 4a, the haplotype “AA” for *xa5* (HB1736) was predictive of the resistant genotypes to PXO86, PXO79, and PXO99 while any genotype having “GG” or “GA” corresponds to susceptibility. For HB3693 flanking the *Xa4* gene, the resistant genotypes to PXO61, PXO71, PXO112, PXO99, and PXO341 mostly exhibited the “TA” haplotype (Fig. 4b).

Among twelve highly significant haplotype blocks, two haplotype blocks (HB814 and HB3460) had SNPs that were not found to be significant in the EMMAX analysis (Additional file 12: Table S6). HB814 was found to be associated with *indica* genotypes’ resistance to PXO112 but SNPs in this haplotype block was not significant in the single SNP association. SNPs comprising HB814 (S2_24907121 and S2_24907123) were 1 Mb away from the most significant SNPs (25,948,468-25,948,531 bp) in chromosome 2 (Additional file 4: Figure S3e and Additional file 6: Table S3). However, looking closely at chromosome 2, the peak started to rise within the region of the HB814. Furthermore, HB3460 was composed of nine SNPs (at 2.9–3 Mb in chromosome 11). Closely inspecting Additional file 4: Figure S3d, HB3460 was the rising peak in the short arm of chromosome 11 which did not reach the threshold of *P*-value < 1 × 10^−5^. This region contained a nodulation-signaling pathway 2 protein (LOC_Os11g06180), an ATOZI1 protein (LOC_Os11g06240), a retrotransposon Ty3-gypsy subclass protein (LOC_Os11g06259) and an integral membrane protein (LOC_Os11g06310).

## Discussion

The population structure of a diverse panel can affect the analysis of genotype-phenotype associations (Zhao et al. 2011). Population structure results in divergence of allele frequencies of the sub-populations. Loci which vary in allele frequencies between sub-populations can be associated with the phenotype when the means of the sub-populations diverge for a particular trait of interest (Zhang et al. 2009b). Principal component analysis of the *indica/aus* panel showed five components that best represent the population structure of the panel. The *indica* genotypes differentiated into three components.

The nine representative strains of eight *Xoo* races used in this study covered a wide range of virulence and prevalence and they successfully distinguished differential resistance across the *indica/aus* panel. Several *indica* genotypes having broad-spectrum resistance comprised mostly irrigated lowland lines and varieties (Additional file 1: Table S1). Other than the IRBB NILs, breeding lines IR09F154, IR08N134, IR 72890-81-3-2-2, IR05N173 and IR09N530 exhibited resistance against seven to nine *Xoo* strains. Most of these genotypes have *xa5* and *Xa4* which were introduced during the breeding process of these varieties. However, the presence alone of the *xa5* gene in IR 72890-81-3-2-2 and IR05N173 would not confer resistance to PXO71 (race4) (Additional file 10: Table S4). Thus, there might be other genes underlying resistance in these genotypes.

Rice genotypes belonging to the group, such as Aus299, Dular, AusBak Tulsi, Ase Pulu Jawa, Aus298, Makalioka 34, Kalimekri 77-5, DV86, Chengri, Aus307, Khaiyan, and DV85 showed broad-spectrum resistance against *Xoo*. Several studies have shown that *aus* genotypes are valuable source of R genes for BB resistance (Sidhu et al. 1978; Khush and Angeles 1999). It is notable that most of the *aus* genotypes (96%) in this study exhibited resistance to both strains of race 9 (PXO339 and PXO349) (Additional file 1: Table S1). Among the *Xa* genes that originated from *aus*, *xa5* is the only gene that confers resistance to race 9 (Iyer and McCouch 2004). To validate if *xa5* solely accounts for resistance to PXO339 and PXO349 in *aus*, alleles of the two SNPs (S5_440644 and S5_453169) overlapping *xa5* were closely inspected (Additional file 10: Table S4). Among the 50 *aus* genotypes, 20 accessions exhibited resistance to the two strains of race 9 but did not have the *xa5* allele (AA allele in DV85 and DV86). Aus299 and Aus 307 exhibited resistance to race 9 and three more races in the absence of the putative *xa5* allele. Thus, other resistance loci in *aus* besides *xa5* confers resistance against strains of race 9.

Our studies confirmed the involvement of seven known *Xa* genes underlying differential resistance. Among the seven *Xa* genes detected in the GWAS, *Xa4*, *xa5* and *Xa21* are known to confer broad-spectrum resistance to *Xoo* (Nino-Liu et al. 2006). SNPs overlapping or linked to the regions of these genes were found to be highly associated with resistance to the expected *Xoo* races. Since *xa5*, *xa13*, *Xa21,* and *xa25* are cloned (Nino-Liu et al. 2006; Liu et al. 2011), the exact physical position of these genes would ascertain that SNPs overlapping these regions could be highly predictive of these genes. Low LD between the regions of the cloned genes and the SNPs detected in GWAS would provide high resolution in tracking these genes in breeding programs.

On the contrary, *Xa4*, *Xa7,* and *Xa14* loci have not yet been cloned. Despite the extensive use of *Xa4* in breeding programs, successful cloning of this gene has not been published. Sun et al. (2003) have narrowed down the *Xa4* locus, most significant SNPs linked to the region of the *Xa4* flanking marker (RM224) overlapped with a rust-resistance protein *Lr21* (LOC_Os11g45620) (Fig. 3). However, Hur et al. (2016) recently fine mapped *Xa4* to a 60 kb region suggesting LOC_Os11g45930, a NBS–LRR-type disease-resistance protein or LOC_Os11g45930, a hypothetical protein as candidates, which lie between SNPs S11_27677853 and S11_27831021, the most downstream of a cluster of 8 significant SNPs overlapping with *Xa4.* Seven significant SNPs were flanking the region of *Xa7* described by Zhang et al. (2009a). Several candidate genes were identified in the two fine mapping studies of *Xa7* (Chen et al. 2008; Zhang et al. 2009a). Among these candidate genes, BTB/POZ and Nramp6, known to be involved in plant resistance (Chen et al. 2008), were within the 84 kb region comprising the SNPs detected in GWAS.

Bao et al. (2010) have mapped the *Xa14* locus between two SSRs having a 300 kb interval. SNPs flanking this mapped region were linked to ATBAG1 (LOC_Os04g52890), OsFBW1-F-box family protein/WD-40 repeat family protein (LOC_Os04g52870) and an ABC transporter (LOC_Os04g52900). These genes were also predicted genes for a blast resistance gene *Pikahei-1(t)* mapped in chromosome 4 (Xu et al. 2008). F-Box and ATBAG proteins were reported to be involved in defense-programmed cell death (Kang et al. 2006; van den Burg et al. 2008) while ABC transporters constitutively induce defense pathways in plants (Chauhan et al. 2015).

Aside from the seven *Xa* genes detected in the GWAS, 33 SNPs clustering on four chromosomes showed strong associations with BB resistance. Among the 8 novel resistance loci identified five appeared to be derived from the *Aus* subpool, underlining the importance of this group as a reservoir for BB resistance donors. *qPXO79_6-2 to -4* cluster on the long arm of chromosome 6 over a range of 1.3 Mb and conferred resistance to race 3B. The discovered markers display a high level of specificity for the *qPXO79_6* loci and could directly be deployed as foreground markers in marker assisted backcrossing (MABC) schemes. At the caveat of introducing linkage drag it would be most feasible to introgress the whole region of 1.3 Mb from a suitable donor within the *Aus* pool used in this study. Likewise the rare Aus specific alleles of *qPXO86_11-1* and *qPXO79/112/341_12-1* could also readily be converted into markers for MABC.

Two loci, *qPXO339/349_9-1* and *qPXO339/349_11-1*, were discovered to confer resistance to race 9A and 9B. New sources of resistance to race 9 of *Xoo* would be valuable. Despite not causing epidemics on rice fields planted with rice varieties having *Xa7*, race 9, particularly race 9b, have persisted at high frequency since the 1990s until present (Ponciano et al. 2004, Quibod et al., 2016). Significant SNPs for both *qPXO339/349_9-1* and *qPXO339/349_11-1*, however, cluster within less than 100 bp, which is too low for reported rice LD. Furthermore, heterozygosity among these SNPs in our panels were very high. Though the corresponding SNP can be found in the 3k genomes, they are not part of the Filtered (4.8 M) or Base (18 M) sets and display similarly high heterozygosity globally, which might indicate wrong mapping due to larger structural variation in the region. Taken together this suggested that both loci should be regarded with reservations as to their accuracy of prediction and utility in resistance breeding.

Nearly half of the identified candidate genes within the 8 novel GWAS peaks code for families that are classically associated with disease resistance such NBS-LRR proteins, NB-ARC domain proteins and receptor-like protein kinases. They are typical R proteins that interact with *Avr* proteins in a gene-by-gene-interaction. In conjunction with the observed high non-synonymous natural variation within the global 3k genomes pool, it makes them high priority candidates worthy of further investigation.

The other half roughly fall into the category of chemical defense through antimicrobial metabolite biosynthesis or transport. ABC transporters have been demonstrated to transport a plethora of secondary metabolites, many of which are toxic to microbes and pests (Kretzschmar et al. 2011). Despite their annotation as peptide transporters PTRs have been shown to transport antimicrobial compounds such as glucosinolates (Nour-Eldin et al. 2012) and plant hormones (Kanno et al. 2012). Likewise, purine permeases transport hormone like secondary metabolites. Serine carboxypeptidases have been shown to act as acyltransferases in terpenoid (Mugford et al., 2009) and phenolic compound (Bontpart et al. 2015) synthesis, many of which act as toxins or deterrents against pests and diseases. Aminotransferases are similarly involved in the biosynthesis of antimicrobial compounds (Ding et al. 2016). Though less specific and often associated with partial resistance only, disease resistance through antimicrobial compounds has been observed to be durable, since resistance breakdown through deleterious *Avr* mutations is not possible. Rather the pathogen has to evolve to catabolize the deterring or toxic compounds. Since candidate gene prioritization was based on the Nipponbare reference and annotation in the respective regions, it can not be ruled out that additional gene models specific to *indica* and/or *aus* were overlooked. Validation of candidate genes will thus depend on comparative genomics among a set of relevant *indica* and *aus* references and transgenic approaches that knock out the R-alleles in the donor background or transfer the R-alleles into recipient backgrounds.

Knowledge about the extent of LD decay is crucial for association analysis and determines its resolution (Remington et al. 2001). LD pattern was quite consistent across chromosomes and decays within 100–200 kb. (Additional file 9: Figure S6). *Indica* genotypes, comprising 69% of the *indica/aus* panel, exhibited a wide range of resistance across the nine *Xoo* strains. Thus, it was of interest to assess the LD, allele frequencies, and haplotypes across genomic regions that are “hotspots” of resistance-associated loci. Lower LD makes mapping resolution higher, allowing isolation of functional variation (Burke et al. 2007). Contrary to the observed average chromosomal LD, regions flanking *Xa* genes have more extensive LD. Among the surrounding regions of *Xa* genes, only the *xa5* region has the least extent of LD of approximately 100 kb which is similar to what Garris et al. (2003) has estimated for the *xa5* region (Additional file 8: Figure S5b). For the distal end of chromosome 11 (26.5–28.9 Mb), the LD extended up to 400 kb. In this region, *Xa* genes such as *Xa3/Xa26* (Yang et al. 2003; Xiang et al. 2006), *Xa4*, and *Xa22*(t) were tightly linked (Wang et al. 2001; Wang et al. 2003). This part of chromosome 11 is known to be a hotspot of resistance genes (The Rice Chromosomes 11 and 12 Sequencing Consortia 2005), and may have been selected for continuously for breeding, explaining a relatively strong LD. Furthermore, the *xa25* region had the most extensive LD, reaching up to ~1500 kb (r^2^ = 0.6). The strong LD in the *xa25* region may be explained by the centromeric location, in which recombination is low (Remington et al. 2001). The relatively stronger LD observed in these regions could be attributed to the relatedness of breeding lines and varieties which composed about 72% of the diverse panel used.

Allele frequency and allele patterns of the 27 SNPs for the seven *Xa* genes detected were characterized to identify markers useful for marker-aided selection. Alleles of the SNPs for the seven *Xa* genes corresponding to expected resistant phenotype have minor allele frequencies, except for SNPs in the *Xa4* region (Additional file 10: Table S4). Homozygous minor alleles predictive of *Xa7-, xa13-*, and *Xa21*-mediated resistance behaved like haplotypes in the *indica* subset even though these SNPs were not detected as haplotype blocks. Table 2 summarizes the SNP markers and alleles predictive of the resistant phenotype mediated by respective *Xa* genes. Among the seven *Xa* genes detected, putative resistant alleles for *Xa4* are the most predominant alleles in *indica* breeding lines and varieties, followed by *xa25* and *xa5*. Genes such as *Xa4* and *xa5* are known to confer broad-spectrum resistance which explains their extensive use in breeding programs. However, it is surprising to note that resistant allele of *xa25* was found to be predominant in breeding lines and varieties. Minghui 63, the donor of *xa25*, is an *indica* rice restorer line (Chen et al., 2002). It can be inferred that breeders have selected for the *xa25* locus unconsciously. Furthermore, the comparison of the R allele frequencies for the 24 SNPs for the seven *Xa* genes in the 1,788 indica genotypes of the 3k genomes (Alexandrov et al. 2015) augments the potential of these SNPs to detect resistance gene alleles (Additional file 11: Table S5).

**Table 2:** Most promising SNPs for marker development

Resistance Gene	SNP	Chromosome	Position	Resistant Allele^a^	Breeding Line and Variety having R Allele (%)^b^
*xa5*	S5_440644	5	440644	A	10
*xa5*	S5_453169	5	453169	A	10
*Xa7*	S6_28100960	6	28100960	A	1
*Xa7*	S6_28158893	6	28158893	G	2
*Xa7*	S6_28160615	6	28160615	G	2
*Xa7*	S6_28171883	6	28171883	C	2
*Xa7*	S6_28173425	6	28173425	A	2
*xa13*	S8_26013849	8	26013849	A	2
*xa13*	S8_26044514	8	26044514	A	2
*xa13*	S8_26044528	8	26044528	C	2
*xa13*	S8_27319974	8	27319974	C	2
*xa13*	S8_27520607	8	27520607	G	2
*xa13*	S8_27536844	8	27536844	C	2
*xa13*	S8_27641374	8	27641374	A	2
*xa13*	S8_27663433	8	27663433	C	2
*Xa21*	S11_21190115	11	21190115	T	4
*Xa21*	S11_21246561	11	21246561	G	3
*Xa4*	S11_27603791	11	27603791	T	60
*Xa4*	S11_27603799	11	27603799	A	59
*xa25*	S12_17305618	12	17305618	A	15
*xa25*	S12_17305619	12	17305619	T	72

^a^ Allele corresponding to resistant phenotype based on 198 indica genotypes

^b^ Percentage of indica breeding lines and varieties having the putative resistant allele

To determine polymorphisms linked to resistance QTLs that were not captured by single SNP associations, haplotype blocks in the *indica* genotypes were also assessed. Of the 3,915 haplotype blocks detected, 12 haplotype blocks were highly correlated with differential resistance, two of which correspond to the *xa5* and *Xa4* genes. HB1736 (*xa5*) and HB3693 (*Xa4*) demonstrated usefulness to predict resistant phenotypes (Fig. 4). For the rest of the haplotype blocks, involvement of the genes to which these haplotypes overlap should be resolved. Thorough analysis of genotypes having haplotypes correlated with resistance is needed to fully realize the value of these genetic variations in BB resistance breeding.

## Conclusion

The results of this GWAS have pinpointed resistance loci conferring differential resistance to the nine representative strains of *Xoo* in the *indica/aus* panel and the *indica* subset. The use of SNPs from genotyping-by-sequencing is a powerful tool to elucidate disease resistance in rice. Differences in the resistance loci detected using two germplasm sets demonstrate the differentiation of loci underlying resistance in terms of the distinct population structure in rice. These results present a wealth of SNPs highly associated with differential *Xoo* resistance that would enable the development of SNP markers for marker-aided selection and tracking of known *Xa* genes. Efficient tracking of *Xa* genes in the breeding pipeline provides breeders insight on which varieties to deploy to specific areas having varying *Xoo* race population. Considering the predominant pathogen race ensures wiser strategies to maintain durability of *Xa* genes deployed in the field. To fully understand the value of these loci in breeding for BB resistance the potentially novel loci identified in this study should be further validated through expression profiling, and development of biparental populations. Moreover, genotypes having no resistance alleles for specific *Xa* QTLs but exhibit high degree of resistance serve as new sources of resistance loci to plant breeders to diversify the genetic base of core breeding sets. The use of *aus* genotypes for a more thorough analysis is recommended to identify potentially novel loci that were not detected in this study.

## Methods

### Selection Of Diverse Panel

An initial diverse panel of 380 genotypes composed of breeding lines, varieties, and landraces was assembled from accessions obtained from the IRRI T.T. Chang Genetic Resources Center and the IRRI Plant Breeding division based on key morpho-agronomic traits and overall field performance. Using 384 SNPs from Illumina BeadXpress system, the 380 genotypes were reduced to 285 genotypes after genetic diversity and population structure analyses using the software packages Powermarker (Liu and Muse 2005), and Structure 2.2 (Pritchard et al. 2000), respectively.

### Leaf Sampling And Genotyping

For reducing the size of the initial diverse panel, leaf samples of the 380 diverse germplasm were collected for SNP genotyping. DNA samples were extracted using the modified CTAB method (Murray and Thompson 1980) and were standardized to 50 ng/μl for Illumina 384-plex BeadXpress SNP genotyping. The 380 genotypes were genotyped using the 384-plex *indica* x *indica* SNP set (VC0013033-OPA) on Illumina BeadXpress system following the Golden Gate Genotyping protocol (Illumina, CA, USA) and allele calling based on the procedure described by Thomson et al. (2012) and Wright et al. (2010).

For the selected panel of 285 genotypes, two to three healthy young leaves were collected from representative tagged plants after the BB resistance screening. The collected leaf samples were lyophilized using the Martin Christ Freeze Dryer at −50° and 0.42 mbar for 48 h, and were ground using the Qiagen Tissue Lyser II. Genomic DNA was extracted using the Qiagen DNeasy Plant Mini Kit. The DNA samples were quantified using the NanoDrop Reader ND8000 and then standardized to 100 ng/μl as required for the preparation of genomic library for GBS.

### Genotyping-by-Sequencing (GBS)

The preparation of the 96-plex genomic libraries for GBS was done in the Institute of Genomic Diversity, Cornell University (Ithaca, NY, USA) following the protocol developed by (Elshire et al. 2011). The purified 96-plex GBS libraries were loaded into the Experion automated electrophoresis station to evaluate fragment sizes. Libraries having no or minimal adapter dimers (fragments of approximately 128 bp in length) and having majority of other DNA fragments between 170 and 350 bp were considered for sequencing. Then, single-end sequencing (86 bp reads) of 96-plex library per flow cell channel were performed on a HiSeq 2000 (Illumina, Inc.) at Cornell University (Ithaca, NY, USA).

Fastq files were processed using TASSEL-GBS analysis pipeline (Version 3.0.147), an extension to the Java program TASSEL (Bradbury et al. 2007). Among the 843,898,432 reads generated from the three lanes of HiSeq, 12,937,086 tags were generated from the TASSEL-GBS Discovery pipeline described by Glaubitz et al. (2014). Tags were aligned to the Nipponbare reference MSU version7 (Kawahara et al. 2013). SNPs were called from aligned tags of at least 10% of the taxa, generating 640,783 SNPs in the hapmap unfiltered file. Post SNP call filtering was also done by retaining SNPs with MAF >0.01 and 80% calling, having 245,669 in the hapmap filtered file.

### BB Resistance Screening

The experiment was carried out in a contained screenhouse facility to prevent the spread of inoculum and was done using RCBD split-plot with nested subplot design. The nine *Xoo* strains were the main plots and in each main plot, a sub-plot of 285 genotypes was nested into early maturing and medium maturing genotypes. The BB resistance screening was performed 45 days after sowing. A total of nine strains representing eight different races of *Xanthomonas oryzae* pv. *oryzae* were used, namely: PX061 (Race1), PXO86 (Race2), PXO79 (Race3B), PXO71 (Race4), PXO112 (Race5), PXO99 (Race6), PXO339 (Race9A), PXO349 (Race9B) and PXO341 (Race10). Inoculation of the rice plants was done through cutting of 1–2 cm of the leaf tip with a pair of scissors dipped in bacterial suspension (Kauffman et al. 1973). The screening was replicated three times over time. For each replicate, five leaves of two plants per entry were inoculated with *Xoo* strains. Lesion lengths were measured 14 days after inoculation. Genotypes having lesion lengths ranging 1–5 cm were rated as resistant (R), 6–10 cm were rated as medium-resistant (MR), 10–15 cm were rated as medium-susceptible (MS), and those having greater than 15 cm were rated as susceptible (S).

The lesion length data generated from the BB resistance screening was averaged across sampling units per entry of each replication. Kolmogorov-Smirnov test for normality of data, analysis of variance of the rice genotypes for the nine strains of *Xoo* using the mixed model analysis of SAS (PROC MIXED), and least square means were generated using SAS version 9.3. LS means generated across three replications were used for association analysis.

### Genetic Diversity, Population Structure, Marker Filtering And Association Analysis

The genetic distance of the 285 genotypes was computed based on Tamura-Nei model implemented in MEGA 6 (Tamura et al. 2013) using 1,193 SNPs (MAF >0.05 and 100% calling) from GBS. In addition, population differentiation *Fst* values were generated from PowerMarker version 3.25 (Liu and Muse 2005) while differentiation based on global unbiased *Fst* (Weir and Cockerham 1984) was calculated using the Hierfstat package implemented in R (Goudet 2005). Principal component analysis was performed using SNP & Variation Suite v8.4 software (Golden Helix, Inc., Bozeman, MT, www.goldenhelix.com).

TASSEL version 3.150 software (Bradbury et al. 2007) was used for manipulating and filtering SNPs for genome-wide association analysis. Initial dataset was filtered based on MAF >0.05 and 95% call rate. A unified mixed-model approach was deployed to account for population structure and familial relatedness (Yu et al. 2006; Price et al. 2006).

Compressed mixed linear model (MLM) was used to analyze association, considering population structure (Q) and relatedness or kinship (K) to reduce spurious associations (Yu et al. 2006). SNP loci with *P*-values < 1 × 10^−5^ were considered significant. Preliminary association results showed ambiguity that residual population structure has not fully corrected by Q + K. Thus, the 37 *japonica*/*aromatic* genotypes were removed from further genome-wide association analysis, retaining 248 *indica* and *aus* genotypes.

Marker-trait association of the 248 genotypes was performed using 40,840 SNPs (90% calling with MAF ≥ 0.05) using SNP & Variation Suite v8.4 software (Golden Helix, Inc., Bozeman, MT, http://www.goldenhelix.com/). Mixed linear models such as Efficient Mixed-Model Association eXpedited Model (EMMAX) (Kang et al. 2008; Vilhjalmsson 2012) and Multi-Locus Mixed Model (MLMM) (Segura et al. 2012; Vilhjalmsson 2012) were used in the analysis Principal component analysis was performed using 10 components, with PC = 5 as the optimal component for the Q matrix. Kinship matrix (K) was generated using Genomic Best Linear Unbiased Predictor (GBLUP) (Van Raden 2008). Q + K matrices were incorporated in the MLM association analysis. Single-locus mixed model (EMMAX) as described by Kang et al. (2010) was done to determine loci associated to resistance against the nine *Xoo* strains assuming all loci have a small effect on the trait. SNPs having *P*-values < 1 × 10^−5^ and FDR corrected values of < 0.05 were considered significant in case they overlapped with known resistance genes (Additional file 5: Table S2). For potentially new loci (Additional file 6: Table S3) a more stringent Bonferroni correction of 0.05 was applied to avoid false positive detection. Furthermore, to determine which loci are cofactors and accounts for large effect on the *Xoo* resistance, MLMM was performed. The MLMM algorithm assumes that multiple loci are associated with resistance, and is a stepwise EMMAX, which re-computes genetic and error variance each step (Segura et al. 2012). Of the 10 steps implemented in MLMM, steps identified to be optimal based on Bonferroni correction was taken account, and *p*-values of loci identified to be cofactors were noted. Since population structure may greatly affect functional variation underlying resistance to BB, separate association analysis of 198 *indica* genotypes using 40,396 SNPs and 3 principal components were carried out.

### Bioinformatics Analysis

The physical positions of BB resistance genes were based on the published position of cloned genes. While for those genes that were not cloned, information of the published flanking markers was used for the ePCR tool of NCBI (Rotmistrovsky et al. 2004) to ascertain the putative position of the genes. To identify genes that overlap and that are closely linked to the significant SNPs, ± 50 bp and ± 150 kb region of the SNP were conservatively considered, respectively. The list of gene annotations was derived from the IRRI Galaxy tool (http://galaxy.irri.org/) linked to the MSU website (http://rice.plantbiology.msu.edu/). In addition, the Rice SNP-Seek Database (Alexandrov et al. 2015) was used to investigate the allele frequencies of significant SNPs in the 1,788 *indica* subset of the 3k genomes and extract non-synonymous SNPs for specific target regions.

### Linkage Disequilibrium, Allele And Haplotype Analysis

Detailed analysis of SNP alleles, linkage disequilibrium (LD) and haplotype blocks for genomic regions associated with differential resistance to nine *Xoo* strains was done using 198 *indica* genotypes. Alleles of significant SNPs flanking/overlapping the seven known *Xa* genes were investigated to determine which allele is predictive of the resistant phenotype to *Xoo*.

Local LD and haplotype analysis were performed using Expectation Maximization (EM) algorithm (Dempster et al. 1977; Excoffier and Slatkin 1995) and the Composite Haplotype Method (CHM) (Weir 1996) on SVS 8.4. Average pair-wise LD (r^2^) was taken account and LD decay (r^2^ < 0.6) was characterized in regions of highly significant SNPs. Furthermore, haplotype blocks were detected on 160 kb window with maximum 30 markers per window, having 50 maximum iterations, and 90% confidence. Haplotype blocks that are correlated to differential resistance were identified using the full model linear haplotype trend regression with Bonferroni adjustment.

## Additional files

Additional file 1: Table S1. Information on the 285 diverse genotypes used in this study. (XLSX 34 kb)

Additional file 2: Figure S1. Phylogenetic and population structure of the 285 diverse panel. a) Radial tree of 285 diverse germplasm based on Tamura-Nei model (MEGA 6). Principal component analysis of b) 285 diverse germplasm c) 248 *indica* and *aus* genotypes, and d) 198 indica genotypes (Golden Helix SVS). (PPTX 318 kb)

Additional file 3: Figure S2. Genome-wide association analysis of bacterial blight resistance to nine *Xoo* strains in 248 genotypes based on Efficient Mixed-Model Association eXpedited Model (EMMAX). Manhattan plots for nine *Xoo* strains (a) PXO61, (b) PXO86, (c) PXO79, (d) PXO71, (e) PXO112, (f) PXO99, (g) PXO339, (h) PXO349, and (i) PXO341. X-axis shows the SNPs along each chromosome; y axis is the − log_10_ (*P*-value) for the association. Significant SNPs are those beyond the red line having *P*-value < 1 × 10 ^−5^. Quantile-quantile plots for nine *Xoo* strains (j) PXO61, (k) PXO86, (l) PXO79, (m) PXO71, (n) PXO112, (o) PXO99, (p) PXO339, (q) PXO349, and (r) PXO341. (PPTX 720 kb)

Additional file 4: Figure S3. Genome-wide association analysis of bacterial blight resistance to nine *Xoo* strains in 198 indica genotypes based on Efficient Mixed-Model Association eXpedited Model (EMMAX). Manhattan plots for nine *Xoo* strains (a) PXO61, (b) PXO86, (c) PXO79, (d) PXO71, (e) PXO112, (f) PXO99, (g) PXO339, (h) PXO349, and (i) PXO341. X-axis shows the SNPs along each chromosome; y axis is the − log_10_ (*P*-value) for the association. Significant SNPs are those beyond the red line having *P*-value < 1 × 10 ^−5^. Quantile-quantile plots for nine *Xoo* strains (j) PXO61, (k) PXO86, (l) PXO79, (m) PXO71, (*n*) PXO112, (o) PXO99, (p) PXO339, (q) PXO349, and (r) PXO341. (PPTX 521 kb)

Additional file 5: Table S2. Significant GWAS SNP hits that are flanking/overlapping with known resistance genes for bacterial blight. (XLSX 92 kb)

Additional file 6: Table S3. Significant GWAS SNP hits that are potentially novel loci for bacterial blight resistance. (XLSX 80 kb)

Additional file 7: Figure S4. Local pair-wise linkage disequilibrium (LD) in GWAS hits overlapping putative novel loci. LD (r^2^) measure was based on Composite Haplotype Method (CHM). Highly significant SNPs corresponding to Additional file 6: Table S3 are highlighted by black arrows. All SNPs in the region including 8 SNPs upstream of the first significant SNP and 8 SNPs downstream of the last significant SNP are shown.in putatively novel peaks are encircled in red. a) SNPs found in chr6: 21.26–21.96 Mb. b) SNPs found in chr6 22.49–23.23 Mb. c.) SNPs found in chr9: 22.16–22.23 Mb. d) SNPs found in chr11: 6.33 M–6.45 Mb. e) SNPs found in chr11: 20.18–20.50 Mb. f) SNPs found in chr12: 25.69–25.76 Mb. (PPTX 2022 kb)

Additional file 8: Figure S5. Linkage Disequilibrium (LD) Decay in GWAS hits overlapping/flanking *Xa* regions. LD (r^2^) measure was based on Composite Haplotype Method (CHM). a.) SNPs flanking *Xa14* (chr 4: 31–32.9 Mb). b.) SNPs flanking *xa5* (chr 5: 200–900 kb). c.) SNPs flanking *Xa7* (chr 6: 26–28.6 Mb). d.) SNPs flanking *xa13* (chr 8: 25–27.9 Mb). e.) SNPs flanking *Xa21* (chr 11: 20.5–22 Mb). f.) SNPs flanking *Xa4* (chr 11: 26.5–28.9 Mb). g.) SNPs flanking *xa25* (chr 12: 13–17.6 Mb). (PPTX 1768 kb)

Additional file 9: Figure S6. Linkage disequilibrium (LD) of adjacent SNPs per chromosome. LD (r^2^) measure of adjacent pairs was based on Composite Haplotype Method (CHM). (PPTX 324 kb)

Additional file 10: Table S4. Genotype matrix of 248 genotypes across GWAS SNPs flanking/linked to *Xa* genes. (XLSX 152 kb)

Additional file 11: Table S5. Comparison of allele frequency of SNPs overlapping/flanking *Xa* genes between GWAS 198 indica genotypes and 1788 indica subset in 3 k genomes. (XLSX 46 kb)

Additional file 12: Table S6. Most significant haplotypes associated with resistance to nine *Xoo* strains. (XLSX 12 kb)

Additional file 13: Table S7. Comparison of *xa5* GWAS SNPs with SNPs derived from 3k Genomes. (XLSX 70 kb)

## Abbreviations

Avr: Avirulence
BB: Bacterial blight
CHM: Composite haplotype method
EM: Expectation maximization
EMMAX: Efficient mixed-model association expedited model
GBLUP: Genomic best linear unbiased predictor
GBS: Genotyping-by-sequencing
GWAS: Genome-wide association study
HPRR: Host pattern recognition receptors
LD: Linkage disequilibrium
MAF: Minor allele frequency
MLM: Mixed linear model
MLMM: Multi-locus mixed model
NIL: Near isogenic line
PAMP: Pathogen-associated molecular patterns
PCA: Principal components analysis
QTL: Quantitative trait loci
SNP: Single nucleotide polymorphisms
*Xoo*: *Xanthomonas oryzae pv. Oryzae*
