# Supplementary material for: Genome-wide Association Analysis Tracks Bacterial Leaf Blight Resistance Loci In Rice Diverse Germplasm
Source: Rice (N Y). 2017 Mar 21;10:8. doi: 10.1186/s12284-017-0147-4 (PMC5359197; doi:10.1186/s12284-017-0147-4)
Supplement: Supplementary file 2 — Phylogenetic and population structure of the 285 diverse panel. a) Radial tree of 285 diverse germplasm based on Tamura-Nei model (MEGA 6). Principal component analysis of b) 285 diverse germplasm c) 248 indica and aus genotypes, and d) 198 indica genotypes (Golden Helix SVS). (PPTX 318 kb) [file 12284_2017_147_MOESM2_ESM.pptx]

## Slide 1
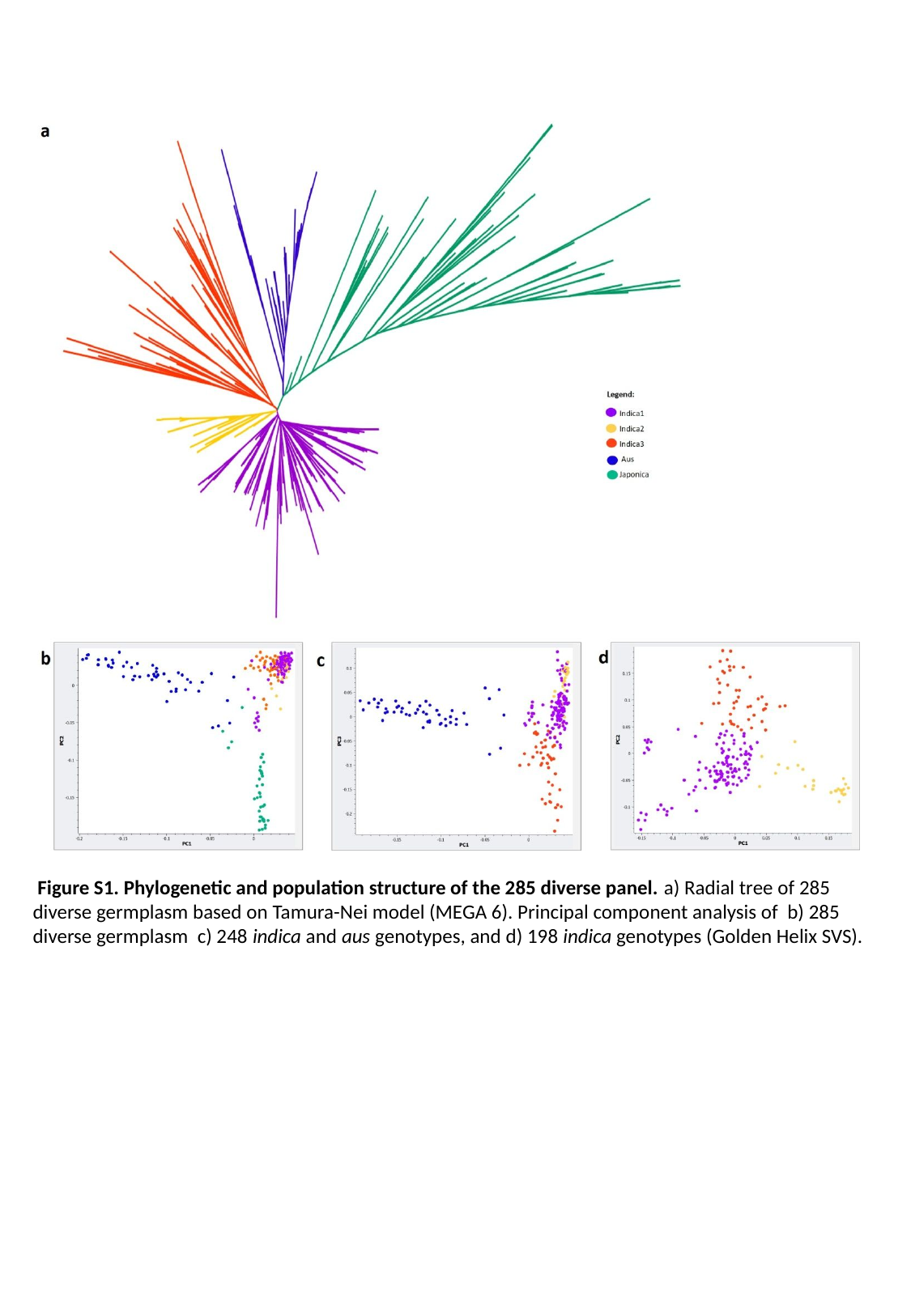

Figure S1. Phylogenetic and population structure of the 285 diverse panel. a) Radial tree of 285 diverse germplasm based on Tamura-Nei model (MEGA 6). Principal component analysis of b) 285 diverse germplasm c) 248 indica and aus genotypes, and d) 198 indica genotypes (Golden Helix SVS).
