# Supplementary material for: Genome-wide Association Analysis Tracks Bacterial Leaf Blight Resistance Loci In Rice Diverse Germplasm
Source: Rice (N Y). 2017 Mar 21;10:8. doi: 10.1186/s12284-017-0147-4 (PMC5359197; doi:10.1186/s12284-017-0147-4)
Supplement: Supplementary file 3 — Genome-wide association analysis of bacterial blight resistance to nine Xoo strains in 248 genotypes based on Efficient Mixed-Model Association eXpedited Model (EMMAX). Manhattan plots for nine Xoo strains (a) PXO61, (b) PXO86, (c) PXO79, (d) PXO71, (e) PXO112, (f) PXO99, (g) PXO339, (h) PXO349, and (i) PXO341. X-axis shows the SNPs along each chromosome; y axis is the − log10 (P-value) for the association. Significant SNPs are those beyond the red line having P-value < 1 × 10 −5. Quantile-quantile plots for nine Xoo strains (j) PXO61, (k) PXO86, (l) PXO79, (m) PXO71, (n) PXO112, (o) PXO99, (p) PXO339, (q) PXO349, and (r) PXO341. (PPTX 720 kb) [file 12284_2017_147_MOESM3_ESM.pptx]

## Slide 1
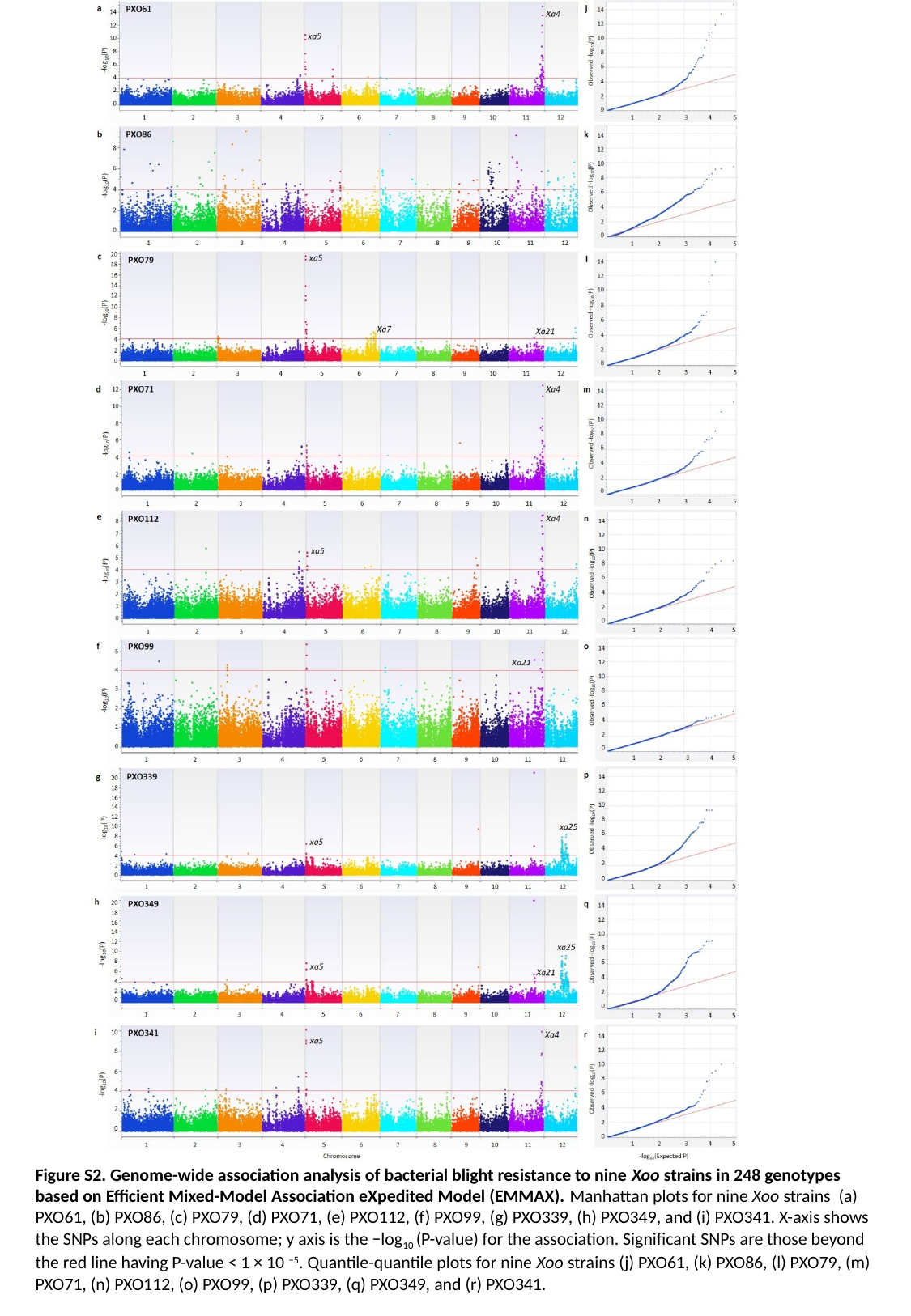

Figure S2. Genome-wide association analysis of bacterial blight resistance to nine Xoo strains in 248 genotypes based on Efficient Mixed-Model Association eXpedited Model (EMMAX). Manhattan plots for nine Xoo strains (a) PXO61, (b) PXO86, (c) PXO79, (d) PXO71, (e) PXO112, (f) PXO99, (g) PXO339, (h) PXO349, and (i) PXO341. X-axis shows the SNPs along each chromosome; y axis is the −log10 (P-value) for the association. Significant SNPs are those beyond the red line having P-value < 1 × 10 −5. Quantile-quantile plots for nine Xoo strains (j) PXO61, (k) PXO86, (l) PXO79, (m) PXO71, (n) PXO112, (o) PXO99, (p) PXO339, (q) PXO349, and (r) PXO341.
