# Supplementary material for: Genome-wide Association Analysis Tracks Bacterial Leaf Blight Resistance Loci In Rice Diverse Germplasm
Source: Rice (N Y). 2017 Mar 21;10:8. doi: 10.1186/s12284-017-0147-4 (PMC5359197; doi:10.1186/s12284-017-0147-4)
Supplement: Supplementary file 7 — Local pair-wise linkage disequilibrium (LD) in GWAS hits overlapping putative novel loci. LD (r2) measure was based on Composite Haplotype Method (CHM). Highly significant SNPs corresponding to Additional file 6: Table S3 are highlighted by black arrows. All SNPs in the region including 8 SNPs upstream of the first significant SNP and 8 SNPs downstream of the last significant SNP are shown.in putatively novel peaks are encircled in red. a) SNPs found in chr6: 21.26–21.96 Mb. b) SNPs found in chr6 22.49–23.23 Mb. c.) SNPs found in chr9: 22.16–22.23 Mb. d) SNPs found in chr11: 6.33 M–6.45 Mb. e) SNPs found in chr11: 20.18–20.50 Mb. f) SNPs found in chr12: 25.69–25.76 Mb. (PPTX 2022 kb) [file 12284_2017_147_MOESM7_ESM.pptx]

## Slide 1
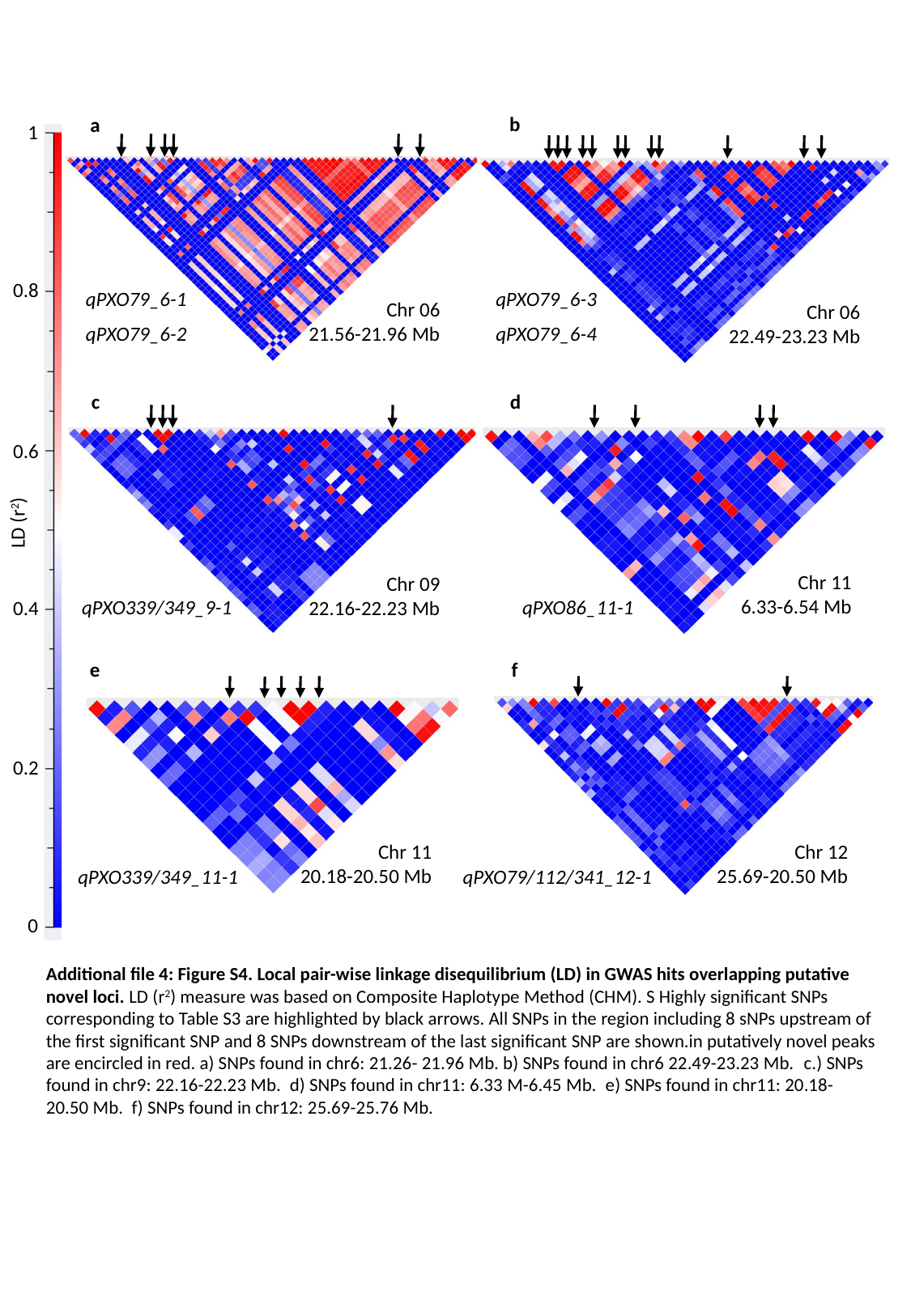

b
a
1
0.8
qPXO79_6-1
qPXO79_6-3
Chr 06
21.56-21.96 Mb
Chr 06
22.49-23.23 Mb
qPXO79_6-2
qPXO79_6-4
0.6
LD (r2)
Chr 11
6.33-6.54 Mb
Chr 09
22.16-22.23 Mb
qPXO339/349_9-1
qPXO86_11-1
0.4
0.2
Chr 11
20.18-20.50 Mb
Chr 12
25.69-20.50 Mb
qPXO79/112/341_12-1
qPXO339/349_11-1
0
d
c
f
e
Additional file 4: Figure S4. Local pair-wise linkage disequilibrium (LD) in GWAS hits overlapping putative novel loci. LD (r2) measure was based on Composite Haplotype Method (CHM). S Highly significant SNPs corresponding to Table S3 are highlighted by black arrows. All SNPs in the region including 8 sNPs upstream of the first significant SNP and 8 SNPs downstream of the last significant SNP are shown.in putatively novel peaks are encircled in red. a) SNPs found in chr6: 21.26- 21.96 Mb. b) SNPs found in chr6 22.49-23.23 Mb.  c.) SNPs found in chr9: 22.16-22.23 Mb.  d) SNPs found in chr11: 6.33 M-6.45 Mb.  e) SNPs found in chr11: 20.18- 20.50 Mb.  f) SNPs found in chr12: 25.69-25.76 Mb.
