# Supplementary material for: Genome-wide Association Analysis Tracks Bacterial Leaf Blight Resistance Loci In Rice Diverse Germplasm
Source: Rice (N Y). 2017 Mar 21;10:8. doi: 10.1186/s12284-017-0147-4 (PMC5359197; doi:10.1186/s12284-017-0147-4)
Supplement: Supplementary file 8 — Linkage Disequilibrium (LD) Decay in GWAS hits overlapping/flanking Xa regions. LD (r2) measure was based on Composite Haplotype Method (CHM). a.) SNPs flanking Xa14 (chr 4: 31–32.9 Mb). b.) SNPs flanking xa5 (chr 5: 200–900 kb). c.) SNPs flanking Xa7 (chr 6: 26–28.6 Mb). d.) SNPs flanking xa13 (chr 8: 25–27.9 Mb). e.) SNPs flanking Xa21 (chr 11: 20.5–22 Mb). f.) SNPs flanking Xa4 (chr 11: 26.5–28.9 Mb). g.) SNPs flanking xa25 (chr 12: 13–17.6 Mb). (PPTX 1768 kb) [file 12284_2017_147_MOESM8_ESM.pptx]

## Slide 1
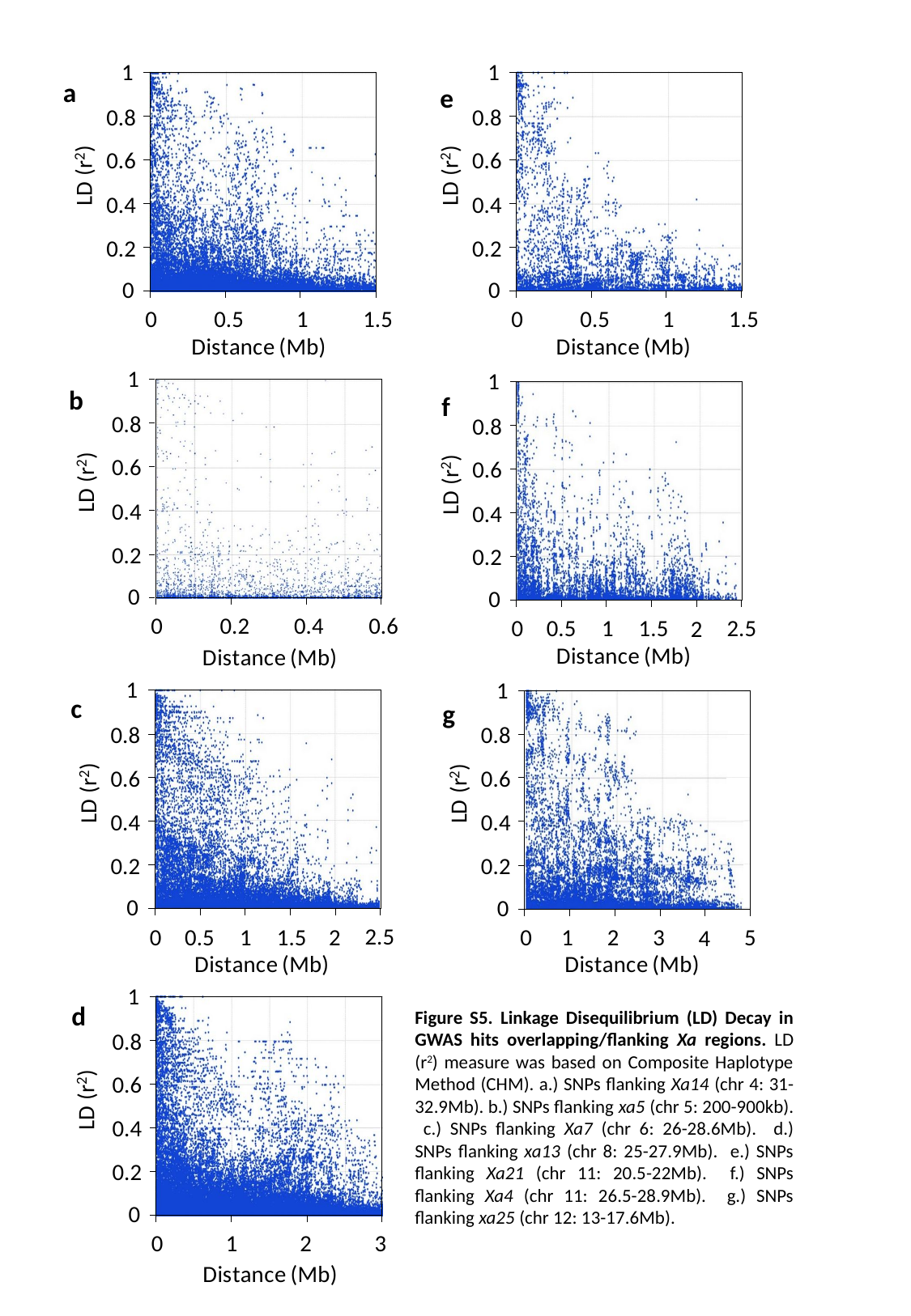

a
e
b
f
c
g
d
Figure S5. Linkage Disequilibrium (LD) Decay in GWAS hits overlapping/flanking Xa regions. LD (r2) measure was based on Composite Haplotype Method (CHM). a.) SNPs flanking Xa14 (chr 4: 31-32.9Mb). b.) SNPs flanking xa5 (chr 5: 200-900kb). c.) SNPs flanking Xa7 (chr 6: 26-28.6Mb). d.) SNPs flanking xa13 (chr 8: 25-27.9Mb). e.) SNPs flanking Xa21 (chr 11: 20.5-22Mb). f.) SNPs flanking Xa4 (chr 11: 26.5-28.9Mb). g.) SNPs flanking xa25 (chr 12: 13-17.6Mb).
