# Supplementary material for: Genome-wide Association Analysis Tracks Bacterial Leaf Blight Resistance Loci In Rice Diverse Germplasm
Source: Rice (N Y). 2017 Mar 21;10:8. doi: 10.1186/s12284-017-0147-4 (PMC5359197; doi:10.1186/s12284-017-0147-4)
Supplement: Supplementary file 9 — Linkage disequilibrium (LD) of adjacent SNPs per chromosome. LD (r2) measure of adjacent pairs was based on Composite Haplotype Method (CHM). (PPTX 324 kb) [file 12284_2017_147_MOESM9_ESM.pptx]

## Slide 1
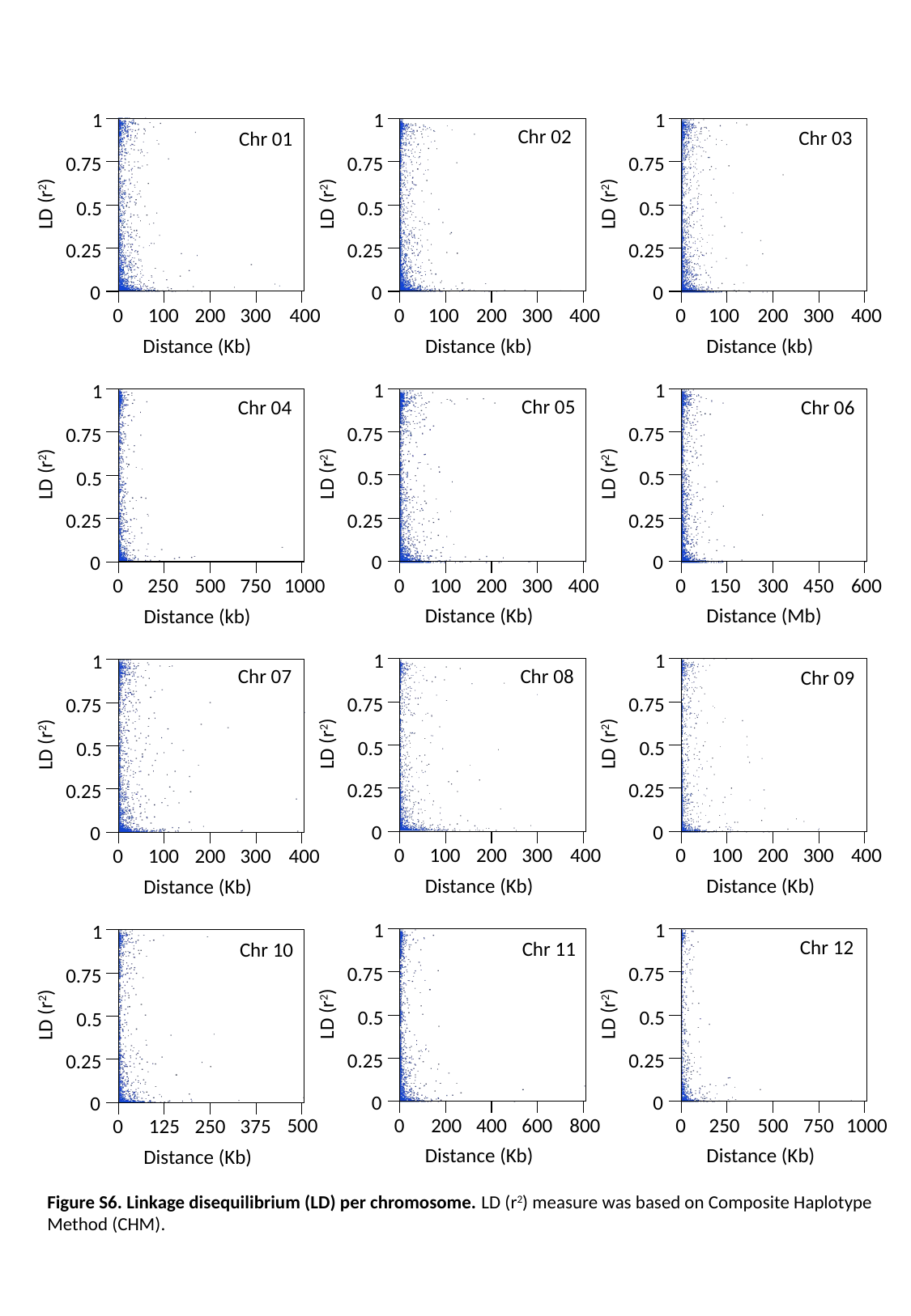

1
0.75
LD (r2)
0.5
0.25
0
0
100
200
300
400
Distance (Kb)
Chr 01
1
0.75
LD (r2)
0.5
0.25
0
0
250
500
750
1000
Distance (kb)
Chr 04
1
0.75
LD (r2)
0.5
0.25
0
0
100
200
300
400
Distance (Kb)
Chr 07
1
0.75
LD (r2)
0.5
0.25
0
500
0
125
250
375
Distance (Kb)
Chr 10
1
0.75
LD (r2)
0.5
0.25
0
0
100
200
300
400
Distance (kb)
Chr 02
1
0.75
LD (r2)
0.5
0.25
0
0
100
200
300
400
Distance (Kb)
Chr 05
1
0.75
LD (r2)
0.5
0.25
0
0
100
200
300
400
Distance (Kb)
Chr 08
1
0.75
LD (r2)
0.5
0.25
0
0
200
400
600
800
Distance (Kb)
Chr 11
1
0.75
LD (r2)
0.5
0.25
0
0
100
200
300
400
Distance (kb)
Chr 03
1
0.75
LD (r2)
0.5
0.25
0
0
150
300
450
600
Distance (Mb)
Chr 06
1
0.75
LD (r2)
0.5
0.25
0
0
100
200
300
400
Distance (Kb)
Chr 09
1
0.75
LD (r2)
0.5
0.25
0
0
250
500
750
1000
Distance (Kb)
Chr 12
Figure S6. Linkage disequilibrium (LD) per chromosome. LD (r2) measure was based on Composite Haplotype Method (CHM).
